# Best Practice Guidance for Digital Contact Tracing Apps: A Cross-disciplinary Review of the Literature

**DOI:** 10.2196/27753

**Published:** 2021-06-07

**Authors:** James O'Connell, Manzar Abbas, Sarah Beecham, Jim Buckley, Muslim Chochlov, Brian Fitzgerald, Liam Glynn, Kevin Johnson, John Laffey, Bairbre McNicholas, Bashar Nuseibeh, Michael O'Callaghan, Ian O'Keeffe, Abdul Razzaq, Kaavya Rekanar, Ita Richardson, Andrew Simpkin, Cristiano Storni, Damyanka Tsvyatkova, Jane Walsh, Thomas Welsh, Derek O'Keeffe

**Affiliations:** 1 Lero, Science Foundation Ireland Research Centre for Software University of Limerick Limerick Ireland; 2 School of Medicine University of Limerick Limerick Ireland; 3 Department of Nursing and Midwifery University of Limerick Limerick Ireland; 4 School of Medicine National University of Ireland Galway Galway Ireland; 5 University Hospital Galway, Saolta, Health Services Executive Galway Ireland; 6 School of Computing and Communications The Open University Milton Keynes United Kingdom; 7 School of Mathematics, Statistics and Applied Mathematics National University of Ireland Galway Galway Ireland; 8 School of Psychology National University of Ireland Galway Galway Ireland

**Keywords:** digital contact tracing, automated contact tracing, COVID-19, SARS-CoV-2, mHealth, mobile app, app, tracing, monitoring, surveillance, review, best practice, design

## Abstract

**Background:**

Digital contact tracing apps have the potential to augment contact tracing systems and disrupt COVID-19 transmission by rapidly identifying secondary cases prior to the onset of infectiousness and linking them into a system of quarantine, testing, and health care worker case management. The international experience of digital contact tracing apps during the COVID-19 pandemic demonstrates how challenging their design and deployment are.

**Objective:**

This study aims to derive and summarize best practice guidance for the design of the ideal digital contact tracing app.

**Methods:**

A collaborative cross-disciplinary approach was used to derive best practice guidance for designing the ideal digital contact tracing app. A search of the indexed and gray literature was conducted to identify articles describing or evaluating digital contact tracing apps. MEDLINE was searched using a combination of free-text terms and Medical Subject Headings search terms. Gray literature sources searched were the World Health Organization Institutional Repository for Information Sharing, the European Centre for Disease Prevention and Control publications library, and Google, including the websites of many health protection authorities. Articles that were acceptable for inclusion in this evidence synthesis were peer-reviewed publications, cohort studies, randomized trials, modeling studies, technical reports, white papers, and media reports related to digital contact tracing.

**Results:**

Ethical, user experience, privacy and data protection, technical, clinical and societal, and evaluation considerations were identified from the literature. The ideal digital contact tracing app should be voluntary and should be equitably available and accessible. User engagement could be enhanced by small financial incentives, enabling users to tailor aspects of the app to their particular needs and integrating digital contact tracing apps into the wider public health information campaign. Adherence to the principles of good data protection and privacy by design is important to convince target populations to download and use digital contact tracing apps. Bluetooth Low Energy is recommended for a digital contact tracing app's contact event detection, but combining it with ultrasound technology may improve a digital contact tracing app's accuracy. A decentralized privacy-preserving protocol should be followed to enable digital contact tracing app users to exchange and record temporary contact numbers during contact events. The ideal digital contact tracing app should define and risk-stratify contact events according to proximity, duration of contact, and the infectiousness of the case at the time of contact. Evaluating digital contact tracing apps requires data to quantify app downloads, use among COVID-19 cases, successful contact alert generation, contact alert receivers, contact alert receivers that adhere to quarantine and testing recommendations, and the number of contact alert receivers who subsequently are tested positive for COVID-19. The outcomes of digital contact tracing apps' evaluations should be openly reported to allow for the wider public to review the evaluation of the app.

**Conclusions:**

In conclusion, key considerations and best practice guidance for the design of the ideal digital contact tracing app were derived from the literature.

## Introduction

### Background

COVID-19 was declared a global pandemic by the World Health Organization (WHO) on March 11, 2020 [1]. COVID-19 has caused the death of over 3.4 million people worldwide as of May 26, 2021 [2]. Most people (as high as 80%) infected by COVID-19 will have no symptoms or mild-to-moderate symptoms [3,4]. Severe illness and death due to COVID-19 are more likely to occur with increasing age and comorbidities such as chronic heart or lung disease [3]. The WHO advise on four key actions to contain COVID-19: social distancing, rapid testing of those with symptoms, tracing of case contacts, and the isolation of suspected and confirmed cases [5]. People with COVID-19 are thought to be most infectious to others within the 2 days preceding symptom onset [3,6]. Onward transmission from individuals in the presymptomatic phase of infection is considered to be enough to sustain the pandemic even if isolation of symptomatic cases occurs [6]. This can be mitigated by stringent social distancing measures, but these come with considerable socioeconomic costs [7].

An effective “test and trace” system is key if the most restrictive social distancing measures such as national “stay at home” orders are to be avoided [8]. Manual contact tracing requires significant human and logistical resources, and its effectiveness depends on the availability and proficiency of contact tracing staff [8,9]. In addition, humans are fallible and prone to recall bias, meaning that not all contacts may be identified reliably in retrospect. It is also not possible in many situations to identify contacts unfamiliar to the case. For contact tracing purposes, the infectious period is considered to be up to 10 days after symptom onset and to begin from 2 days before symptom onset or if the person is asymptomatic from 2 days before testing [10,11]. The incubation period for COVID-19 can be up to 14 days (and longer in 5% of cases) [12], meaning that not all contacts are captured by this definition. COVID-19 has a serial interval as short as 3.2 days; therefore, contact tracing and quarantine of contacts must be rapid to disrupt transmission chains [13].

A digital contact tracing app (DCTA) is an app that can detect and trace other app-carrying individuals who have had contact with one another that would risk COVID-19 transmission if one were to be infected. Early in the pandemic, DCTAs were seen as a potentially innovative solution to contain COVID-19 by augmenting the effectiveness of manual contact tracing [14]. DCTAs could disrupt transmission chains by rapidly identifying secondary cases prior to the onset of infectiousness and linking them into a system of quarantine, testing, and health care worker case management [8,15-17]. A COVID-19 modeling study from the United Kingdom estimated that if a DCTA were used by 56% of the population, then the reproductive value of the virus could be reduced below 1.0, controlling the disease [18]. By October 13, 2020, there were 120 DCTAs in 71 countries [19], and within months of digital contact tracing use, some key challenges became evident.

### Global Digital Contact Tracing App Deployment

On March 20, 2020, Singapore became the first country in the world to launch a national DCTA, TraceTogether [20]. TraceTogether was downloaded by over 1.1 million users within a month of launching, despite having technical limitations [21]. It required Apple iPhone users to have the app open in the foreground and caused significant battery drain [21]. In South Korea, an extensive electronic surveillance system was used. GPS-enabled location tracking, closed-circuit television recordings, and credit card transactions were used to aid contact tracing [22]. How these data were used by health authorities to warn others of potential exposure to COVID-19 may have breached the privacy of those infected and contributed to a growth in social stigma associated with the disease [23]. In Israel, a network-based mass surveillance system using mobile phone GPS technology was launched on March 16 to identify COVID-19 case contacts [24,25]. Authorities in Israel reported that, after 1 month of surveillance, 36.8% of COVID-19 cases notified were identified using the surveillance system [25], although the system did have a false-positive detection rate of 5% [25]. Significant privacy concerns were raised by opponents of Israel’s surveillance system, but ultimately, the supreme court ruled in favor of its use provided it was supported by primary legislation [24-26]. In Norway, a DCTA that used Bluetooth Low Energy (LE) and GPS location tracking was launched on April 16 and was downloaded 1.6 million times [27]. However, the National Institute of Public Health was forced to abandon the app after data protection authorities deemed there was no evidence of its effectiveness to justify location data collection [27]. Norway remained without a DCTA for several months thereafter [27]. Qatar mandated the use of its “Ehteraz” DCTA on May 22, but subsequently, it was discovered that it left the health status and location data of over 1 million users vulnerable to cyberattacks [28]. On July 7, the Republic of Ireland (ROI) launched a national DCTA called COVID Tracker, which is actively used by 1.3 million people, 34% of those older than 16 years nationally [29]. However, a service update in early August caused rapid battery depletion and heat issues for some users [30,31]. This was the primary cause of negative feedback for COVID Tracker [32]. In the 5-day period after this update, 152,656 uninstalls were registered with 29,049 returning users recorded [33]. As of May 28th 2021, eleven months after its release, COVID Tracker has been used by 15,742 people with COVID-19 to send contact alerts to 24,436 users [29]. In the United Kingdom, the National Health Service (NHS) COVID-19 app, launched on September 24, had been erroneously notifying users they were close contacts but provided no further instructions [34,35]. The international experience of DCTA use during the COVID-19 pandemic demonstrates how challenging their design and deployment are. This formed the basis of this literature review, which aims to derive and summarize best practice guidance for the design of the ideal DCTA (IDCTA).

## Methods

A collaborative cross-disciplinary approach (Multimedia Appendix 1 [32,36-39]) was used to derive best practice guidance for designing the IDCTA. The cross-disciplinary team included specialists from computer science, engineering, clinical medicine, medical technology, and psychology. A scoping review to identify considerations described in the emerging literature on DCTAs was conducted (Multimedia Appendix 2 [8,14,15,36,40-42,44]). After the key considerations were identified and agreed upon by the cross-disciplinary team, a detailed evidence synthesis for each was constructed by author JOC and refined through a review and feedback cycle involving a subgroup of the cross-disciplinary team. The cycle of review and feedback was repeated until there was cross-disciplinary agreement that all feedback had been adequately addressed. The product of this process was then presented to the wider cross-disciplinary team for further discussion, from which best practice guidance for the design of the IDCTA was derived through a review and feedback cycle.

To construct the evidence synthesis, a literature search was conducted using Ovid MEDLINE and Epub Ahead of Print, In-Process, and Other Non-Indexed Citations, Daily and Versions. Free-text terms and Medical Subject Headings search terms were used (Multimedia Appendix 3). The WHO Institutional Repository for Information Sharing [45] and the European Centre for Disease Prevention and Control (ECDC) publications library [46] were searched. The gray literature search (Multimedia Appendix 4) included manually searching the websites of DCTAs, media sites, and health protection authorities including but not limited to the Centers for Disease Control and Prevention (United States of America), Public Health England, Health Protection Surveillance Centre (ROI), Robert Koch Institute (Germany), and the Norwegian Institute of Public Health. Included articles had to describe aspects of developing or deploying a DCTA. Peer-review publications, modeling studies, cohort studies, randomized trials, technical reports, white papers, and media reports were eligible for inclusion. The references of included articles were also searched to identify other eligible literature. Both English and non-English articles were included, and Google Translate was used to translate non-English articles.

## Results

### Key Considerations

From the scoping review (Multimedia Appendix 2), the cross-disciplinary team identified and agreed upon six key considerations for best practice guidance when designing the IDCTA: (1) ethical considerations, (2) user experience considerations, (3) privacy and data protection considerations, (4) technical considerations, (5) clinical and societal considerations, and (6) evaluation considerations.

**Figure 1:**
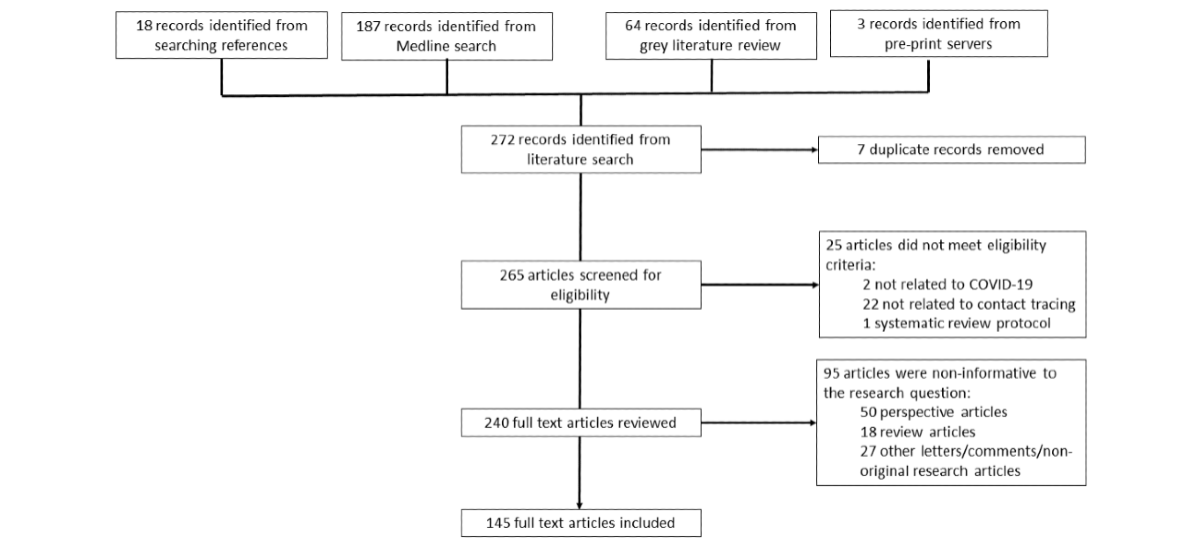
Literature search flow diagram.

The outcome of the literature search shown in Figure 1 and Multimedia Appendix 5 [14-17,22,23,25,26,32,34,37, 38,40-42,44,47-173] contains a description of the included studies and their source (indexed literature search, gray literature, references search).

For each consideration, best practice guidance for the design of the IDCTA as derived from the literature by the cross-disciplinary group is summarized.

### Ethical Considerations

On December 10, 1948, the United Nations General Assembly adopted the Universal Declaration of Human Rights, which included the right to health [174]. Digital contact tracing can be viewed as states using their available resources to protect people’s right to health. However, it may also be viewed as states interfering with other human rights enshrined in the Declaration, such as the right to human dignity; nondiscrimination; equality; privacy; access to information; and the freedoms of association, assembly, and movement [47,174]. Such interference to protect health may be ethical if it is adherent to criteria defined by the Siracusa Principles in 1985 [175]. These criteria are that interferences should have a legal basis, further a legitimate objective of common interest, not disrupt democratic processes, and not be deployed in an arbitrary or discriminatory way [175]. These form the most rudimentary ethical design considerations for the IDCTA.

There are many frameworks through which the ethics of DCTA’s use can be considered in greater detail. Upshur [48] described four guiding principles when considering whether a public health intervention is ethical (the harm principle, the principle of least restrictive means, the reciprocity principle, and the transparency principle). Childress et al [49] provide a set of five justificatory conditions (effectiveness, proportionality, necessity, least infringement, and public justifications) necessary for a public health intervention to interfere with individual liberties. Kass [50] described an ethical framework for public health interventions based on an assessment of the effectiveness, potential harms, alternative interventions and options for harm interventions of the intervention, and whether it is an equitable intervention. However, the complexity and lack of clarity of the ethical issues surrounding the relatively recent advent of digital contact tracing in the context of what was a new rapidly evolving global pandemic have necessitated frameworks that specifically address ethical considerations in digital contact tracing [15,51-56]. An overview of the key ethical considerations presented in these frameworks is provided in Multimedia Appendix 6 [15,51-56]. There is considerable agreement between the frameworks and among early guidance from the European Commission [57] and digital technology expert groups [14,58] about the considerations necessary to design and deploy DCTAs ethically, which broadly can be described as proportionality, voluntariness, transparency and trustworthiness, and equity.

Proportionality, that is, ensuring the intervention is a proportionate response to the public health threat, defines the ethical limits of other aspects of the IDCTA, such as its clinical and societal use and its interference with privacy and data protection rights [14,58,59,176]. There is no doubt that COVID-19 is a significant threat to public health, as evident even from early reports [177]. However, to determine if DCTAs are a proportionate response to this threat, an assessment of their potential benefits and risks is required. As a key component of the WHO-advised strategy to counter COVID-19 [5], any intervention that improves the effectiveness of contact tracing is of benefit to public health during the pandemic. However, high-quality evidence that DCTAs are effective in doing this is lacking [60,61]. Randomized controlled trials are the gold standard when evaluating the effectiveness of interventions, but for DCTAs, they may be logistically challenging and costly to design [62]. Augmenting manual contact tracing through nondigital means has proved problematic, particularly in Western societies [63,178]. In the absence of other alternatives to augment manual contact tracing and given the uncertainty regarding DCTA effectiveness, a key feature of the IDCTA should be harm minimization [53]. As summarized in Textbox 1, this includes minimizing the risk to personal data and privacy, and minimizing the risk of the false characterization of contact status.

**Potential risks of digital contact tracing apps.**

**Data protection**

- Failure to protect personal data from misuse [64,65]

**Privacy**

- Loss of personal privacy with no personal or societal benefit [59,61,66,176]

**Resources**

- Misuse of limited financial and human resources on an ineffective intervention [67,68]

**Clinical**

- False-positive characterization of contact status (may result in unnecessary quarantining and anxiety) [69,70]
- False-negative characterization of contact status (may result in further onward disease transmission) [69,70]

**Public engagement**

- Loss of trust in public health authorities and public health measures [14,56,66]

The IDCTA should be voluntary and consent-based according to the WHO and ECDC ethical guidance on DCTAs, and this view is also prevalent in the academic literature [40,51-55]. For voluntary DCTAs, ensuring transparency and trustworthiness is important to maximize population penetration [52,53,71-79]. Transparency can be achieved by making the underlying DCTA algorithms open source [52,80,81], and this is established as best practice [82,83]. Similarly, an explanation of the risk prediction algorithm used should be publicly available for scrutiny [52] as has been done by Germany’s Corona-Warn-App [179]. The data processed by the IDCTA, including the type, purpose, and duration of data storage, should be presented in an accessible, transparent way for all DCTA users [52,80,81]. Trustworthiness (how trusted a DCTA is by the target population) depends not only on transparency, robust data protection, and privacy preservation [37,72,84,85] but also on having published guiding ethical principles [86], a defined timeline and plan for DCTA evaluation, a defined published criteria for DCTA deactivation, and a DCTA independent oversight committee that has representation from civic society and the public [15,51,52].

DCTA availability and accessibility must be equitable, and they should not be used in a discriminatory way [52,56,66,87,175]. This is important not only from a human rights perspective but also from an effectiveness perspective because the success of a DCTA depends on factors such as user penetration within the general population [88] and in high-risk population groups [15,89]. The IDCTA should be disseminated free of charge, so it is accessible by all societal groups but, in particular, those disproportionately affected by COVID-19 such as older adults, people of lower socioeconomic class, and ethnic minorities [51,52,56,61,90,91]. Smartphone technology may be inaccessible to people of low incomes or those with limited digital literacy [92]. In Singapore, Bluetooth-enabled contact tracing tokens have been distributed to older adults who were less likely to be smartphone owners [93]. This practice should be encouraged, and alternatives such as free smartphones or monofunctional digital contact tracing devices should be deployed in parallel to groups who may not otherwise have access to DCTAs [14,94,95].

### User Experience Considerations

The IDCTA should be designed so that it synchronizes two independent environments, that of public health authorities and that of end users. DCTA user experience considerations can be thought of as those relating to universality and those relating to user engagement. Multimedia Appendix 7 [29,32,36,38,39,42,69,96-104,106,107,110-118,180-182] provides an overview of the key academic literature [75,96-105], gray literature [106,107], regulations [42,108-111], guidelines [112,113,180], and assessments of existing DCTAs [29,32,38,114-118] that support these recommendations.

To support a more holistic approach to the design of the IDCTA, the concept of universality allowed the cross-disciplinary team to identify a series of dimensions to be taken into account, such as accessibility, minors as users, cultural universality, content, availability, and maintenance and frequency of upgrades with the aim of better accommodating different users’ needs, including minors, older adults, people with chronic disease, and those with various forms of disability, so that accessibility and inclusiveness can be ensured [42]. In keeping with this holistic approach to the design of the IDCTA, the interface elements should enable multimodal interaction (eg, supported by voice control) with contents that are available in different languages. Additionally, jargon should be avoided. How well the dimensions of universality are incorporated into the design of the IDCTA will affect not only its population penetration and continued use but also its interoperability across borders. Population penetration will depend on the prevalence of smartphones and operating systems in use among the target population that support the chosen DCTA technology [75,105]. Trade-offs between accuracy and availability will need to be assessed so that it is available on the widest range of smartphones and operating systems possible (eg, ultra-wideband is accurate but not widely available [183,184] and Bluetooth LE is less accurate but more widely available [119,120,185]). This must be done while also supporting the various screen sizes and resolutions of the widest range of smartphones in use among the population. The IDCTA should be conceived as open-ended with frequent updates, ongoing support, and constant maintenance.

Regarding user engagement, nine key aspects were identified from across the literature that could help improve engagement: performance feedback, helpfulness, public health measures, educational information, personal information, personalization and control, time and human effort, flexibility or multimodality, and multitasking. Based on these aspects, user requirements that could increase engagement are evident. Engagement could be potentially enhanced by enabling the user to contact their case health care worker should they have questions regarding their COVID-19 diagnosis. Engagement could also potentially be enhanced by allowing users to identify areas where the incidence of COVID-19 infection is high that they may wish to avoid or settings where the risk of contracting COVID-19 when exposed may be highest (eg, public transport routes known to be frequently crowded). Dynamic, consistently updated information on confirmed cases, testing sites, vaccination sites, government restrictions, and preventive strategies could enhance user engagement by making the benefit of using the app more apparent to the users and integrating it with the wider public health information campaign as part of the national COVID-19 response. However, the amount of information presented should not be overwhelming for users. Graphic representation of these data may also be beneficial (eg, visualization may summarize the number of cases or close contacts being reported per day or week). By conveniently providing useful information on the DCTA, it has the potential to engage and help users long-term to protect themselves against COVID-19. The IDCTA should also enable the end user to tailor the app to their particular needs to enhance user engagement. For example, users might find it beneficial to personalize which notifications they receive or to temporarily deactivate the contact tracing function [121].

### Privacy and Data Protection Considerations

Privacy and data considerations of DCTAs are dependent on what their functional requirements are. DCTAs need to maintain a contact log, generate a contact alert, and link users with the test and trace system. The IDCTA needs to perform these functions while respecting individual privacy rights and adhering to data protection regulation [14,22,37,40-42,52,56, 69,72,74-76,84,122-124]. The European Charter of Human Rights Article 8 states that individuals have a right to respect for private life, but interference with this right can occur if it is deemed necessary, proportionate, and in accordance with the law [186]. The IDCTA should follow the foundational principles of privacy by design, a widely used approach in systems engineering characterized by proactive rather than reactive measures, and this approach to digital contact tracing is supported by the European Data Protection Board (EDPB) [125]. The collection and use of personal data are protected by several regulations in the European Union, such as the European General Data Protection Regulation (GDPR) Act 2016 and the ePrivacy Directive 2002 [126,187]. Article 6 of the GDPR states that processing of data is lawful if it is “necessary to protect the vital interests of a person” and if it is “necessary for the performance of a task carried out in the public interest” [187]. Contact tracing of infectious diseases is lawful because it is necessary to protect case contacts, and epidemic containment is certainly carried out in the public interest, a view which is supported by the WHO and ECDC [5,40]. Data concerning health such as one’s COVID-19 infection or contact status are considered “special data” as described in Article 9 of the GDPR [187]. The processing of “special data” is permissible only where “processing is necessary for reasons of substantial public interest,” which shall be “proportionate to the aim pursued” and respects “the essence of the right to data protection” [187]. More specifically, special data may be processed if it is “necessary for reasons of public interest in the area of public health, such as protecting against serious cross-border threats to health or ensuring high standards of quality and safety of health care” [187]. As further explained in recital (46) of the GDPR, the processing of personal data should also be regarded to be lawful where it is necessary to protect an interest that is essential for the life of the data subject or that of another natural person, for instance, when processing is for monitoring epidemics [127]. Evidently, data collection and processing to facilitate contact tracing during an epidemic to prevent further disease transmission and death is permissible. There are limits to this as defined in Article 5 of the GDPR, which sets out seven key principles related to the processing of personal data (Textbox 2) [128].

**Principles of data protection.**

**Lawfulness, fairness, and transparency**

- Lawfulness: Processing of personal data carried out by a controller must have a legal basis under the General Data Protection Regulation.
- Fairness: Processing of personal data must be fair toward the individual whose personal data are concerned and avoid being unduly detrimental, unexpected, misleading, or deceptive.
- Transparency: Controllers must provide individuals with information regarding the processing of their personal data in a format that is concise, easily accessible, and easy to understand.

**Purpose limitation**

- Personal data must be collected for specified, explicit, and legitimate purposes.

**Data minimization**

- Personal data that are collected and processed should be adequate, relevant, and limited to what is necessary for the purposes for which they are processed.

**Accuracy**

- Personal data that are collected should be accurate and, where necessary, kept up to date.

**Storage limitation**

- Controllers must hold personal data, in a form that permits the identification of individuals, for no longer than is necessary for the purposes for which the personal data are processed.

**Integrity and confidentiality**

- Personal data must be processed by controllers only in a manner that ensures the appropriate level of security and confidentiality for the personal data using appropriate technical or organizational measures.

**Accountability**

- Controllers are responsible for, and must be able to demonstrate compliance with, the other principles of data protection.

To be lawful, there must be a legal basis on which the data are processed [109]. For example, in the ROI, a DCTA was introduced on the legal basis set out under section 7 of the Health Act 2004, which states that health authorities should use its resources to protect the health and welfare of the public [185]. Use of the IDCTA should be voluntary, and this should be included in legal frameworks when legislating for its use [40,51-55]. To ensure accountability, the controller of the DCTA should be clearly defined and the EDPB suggest this could be national public health authorities [41].

DCTA contact logs should adhere to the principles of privacy by design and data minimization by collecting only an anonymized identifier unique to each contact event [41]. This means the IDCTA should not record the name, age, sex, ethnicity, or address of the contact nor should it record the time or location of the contact event [41]. However, privacy needs to be embedded into the DCTA design without diminishing functionality as much as is possible [125]. To enable risk stratification of the contact event, the IDCTA should record the day of the contact event, as is done by the Corona-Warn-App [179]. Although collection of location data is recommended against by the EDPB, in the ROI and the United Kingdom, there is some evidence that the majority of people do not have an objection to its use by DCTAs in the context of an epidemic [37,121,129]. Although this may vary between countries, where location tracking is deemed a proportionate response to the scale of the epidemic, the IDCTA should log the location of the app user locally [121] but not that of their contacts in keeping with privacy by design principles [125]. The principle of purpose limitation [128] would dictate that both contact and location tracking logs should be collected only for the purpose of COVID-19 contact tracing. How a contact event is recorded by a DCTA must be accurate; otherwise, there is the potential for large-scale misclassification of contact events as occurred with the UK NHS COVID-19 app [34,35]. Therefore, field studies that validate the accuracy of the app should be performed and published as has been done for some DCTAs [130]. In the ROI, independent assessments of COVID Tracker have been performed and published, and this practice should be encouraged [131,132]. Contact logs should be maintained for 14 days, the incubation period of COVID-19 [3], to adhere to the principle of storage limitation.

When a DCTA user is confirmed to have COVID-19, an exposure notification system is necessary to enable them to alert their contacts. To ensure data collected are accurate, it has been suggested that COIVD-19 cases have their status verified before they can use the exposure notification system to prevent misuse [133,134]. Verification should preferably be automated [135]. To ensure integrity and confidentiality, the EDPB [41] and European Union eHealth Network [42] recommend that contact log processing follow a decentralized privacy preserving protocol (ie, processing of contact logs to match those of the user’s contacts with those of cases that occurs on the user’s device). The principle of privacy by design [125] would dictate that contact alerts should be generated from the contact’s DCTA as opposed to being sent from the case’s DCTA. The contact alert, to be adherent to the principle of data minimization, should not contain the cases’ personal information such as name, age, sex, or ethnicity nor should it contain the time or location of the contact event [41]. For example, contact alerts generated by COVID Tracker in the ROI inform users “Close Contact Alert: The app has detected that you have been in close contact with someone who has tested positive for COVID-19” [29]. When a contact alert is generated, the DCTA user should be able to contact public health authorities through the app [32], or they should be provided with a number to contact health authorities on.

In some countries such as South Korea and Israel, interference with individual privacy rights was deemed to be a necessary proportionate response to COVID-19. However, Western societies particularly value privacy [37,136]. Maximizing population penetration of a voluntary DCTA in these societies will require health authorities to convince target users that their privacy will be protected [68,72,77,137]. The nature of data collected, whether it be proximity data, location data, or both, hinges on whether it is deemed to be proportionate to the aim pursued. The EDPB state that DCTAs should rely on proximity and not location data [41]. Many countries such as the ROI, the United Kingdom, and Germany have developed DCTAs that record proximity using Bluetooth LE [29,34,179]. However, from the experiences of Israel and South Korea, location tracking may be a key feature of effective digital contact tracing. Tracking location may be useful to identify previously unknown settings where transmission is occurring, allowing for public health authorities to take proactive action to prevent further transmission [25,138,139]. However, the privacy risks are significant, and misuse of location data can be harmful to public trust in health authorities [23]. There is a need for public engagement mechanisms in each country to define by consensus what the limits of a proportionate response to COVID-19 are. Where location tracking is used, it should be an opt-in feature [121] because invasion of personal privacy can be a significant deterrent to downloading and using a DCTA, and the use of geolocation data has recently been the cause of privacy losing events associated with app use [85,140].

In May 2020, Google and Apple collaborated to create an application programming interface (API) [141]. An API is a *Lego block* on which governments can build a DCTA. The DCTA (interface, data collection, public health information), server (epidemiological dashboard, diagnosis verification), and server relay (for 14 days of the case’s cryptogenic keys) are supplied by the public health authority [141]. The Google/Apple API works on Android 6 and iOS 13 forward [141]. Building a DCTA on this API requires a series of measures to protect individual privacy. There should be a requirement for explicit user consent, anonymity of all users to each other, and allowing users the choice of how much personal information they share [81,141]. Google and Apple control which DCTAs use the API and for how long the API is operational in a given region [141]. There should be an agreed timeline and criteria for when the API and DCTA infrastructure is to be dismantled [76,121,124,141] because there are significant privacy concerns that technology companies and governments could use DCTAs to enable greater surveillance after the pandemic [26,37,65,66,76]. Using an API comes with the risk that personal data may be misused or processed unlawfully. Google has a record of not adhering to data protection regulations and the principles of data protection [188]. There are also ongoing concerns regarding Google’s lawful and transparent use of location data [189]. The entry of private corporations into pandemic response may create a dependency on them to deliver public health necessities, global health policies, and result in an accumulation of decision-making powers across multiple aspects of society and subversion of democratically elected governments [59,176]. Despite concerns surrounding the role of private corporations in digital contact tracing, the number of app downloads was high [37,190]. This may be explained by the phenomenon known as the privacy paradox, whereby people express concerns regarding sharing personal information, but their behavior is incongruent with the concerns they express [142].

### Technical Considerations

For the IDCTA, the choice of which technology to use to detect contact events will be influenced by its cost, energy use, accuracy, availability, accessibility, adherence to data protection regulation, and by how it effects privacy preservation and overall DCTA effectiveness [14,17,37,40,42,52,61,69,76,141]. These in turn influence DCTA population penetration. Potential technologies include ultra-wideband technology, Wi-Fi, Bluetooth LE, ultrasound, and GPS (Table 1). Ultra-wideband technology is an ideal technology for proximity detection. It is a low cost, low energy use technology that can measure highly accurate spatial data both indoors and outdoors, with an ability to discriminate distances of 10 to 30 cm [143,183,184]. With regard to privacy, it is considered more secure than Bluetooth LE [143]. However, smartphones equipped with this technology are not yet in common use [184]. A DCTA that uses Wi-Fi would be limited by range and difficult to make ubiquitously available. According to the EDPB and the European Union eHealth guidance, Bluetooth LE proximity detection should be used because it maximizes privacy preservation and is widely available, which are important to maximize population penetration [41,42,119]. However, population penetration will also rely on belief in the accuracy of the DCTA to detect contact events [79,105,144,145]. DCTAs that use Bluetooth LE alone may not have adequate accuracy [70,132,146,147,185]. GPS location tracking may be less accurate indoors or in multistory buildings as compared with Bluetooth LE [44,148]. Published studies validating the accuracy of these DCTA technologies are lacking (Table 1). Combining ultrasound technology with Bluetooth LE may improve accuracy by reducing the number of false-positive contacts identified [116,130]. In the absence of widely available ultra-wideband technology, this represents the best compromise on privacy preservation, accuracy, and availability for the IDCTA. However, this may not be applicable across all countries because the choice of technology will depend on the prevalence of compatible smartphones in use in the population and how valued personal privacy is among the population.

**Table 1:** Potential DCTA technologies and the implications of their use.

DCTA^a^ technology	Bluetooth LE^b^	GPS-enabled geolocation tracking	Bluetooth LE and ultrasound	Ultra-wideband
Accuracy^c^	Accuracy reported as 72%^d^ (distance threshold not reported) and 79% (distance threshold 1.5 m); although, independent studies did not reproduce these results [70,147,185].	Accurate to within 4.9 m, but concerns that GPS location tracking for COVID-19 contact tracing not feasible due to limited accuracy [149,191]	Accuracy reported as 55% (distance threshold ≤6 foot) and accuracy reported as 99.6% (distance threshold ≤12 foot) [130]	Highly accurate [143]
Effectiveness in augmenting manual contact tracing	Limited evidence to suggest effectiveness [150,151]	Limited anecdotal evidence to suggest effectiveness [25,138]	Insufficient evidence found to suggest effectiveness	No instances of ultra-wideband–enabled DCTAs found in the literature.
Energy use	Less than GPS [148]	More than Bluetooth LE [148]	Not reported	Low energy use [143]
Accessibility and availability	Widely available [119,120]	Widely available [120]	Widely available but less so than Bluetooth LE or GPS	Not widely available [143,183,184]
Adherence with principle of privacy preservation	Highly adherent (records only proximity)	Less adherent (records location, which is potentially identifiable)	Adherent^e^ (records only proximity)	Highly adherent [143] (records only proximity).
Adherence with principles of data protection	Adherent	Interferes with the principle of data minimization	Adherent	Adherent

^a^DCTA: digital contact tracing app.

^b^LE: Low Energy.

^c^(True positives + true negatives) / total number of tests.

^d^COVID Tracker Ireland reported being able to accurately identify 72% of close contacts, although field studies supporting this claim have not been published.

^e^Perception that it has the potential for misuse of audio data [152], but this is not the case according to proponents of this technology [153].

**Figure 2:**
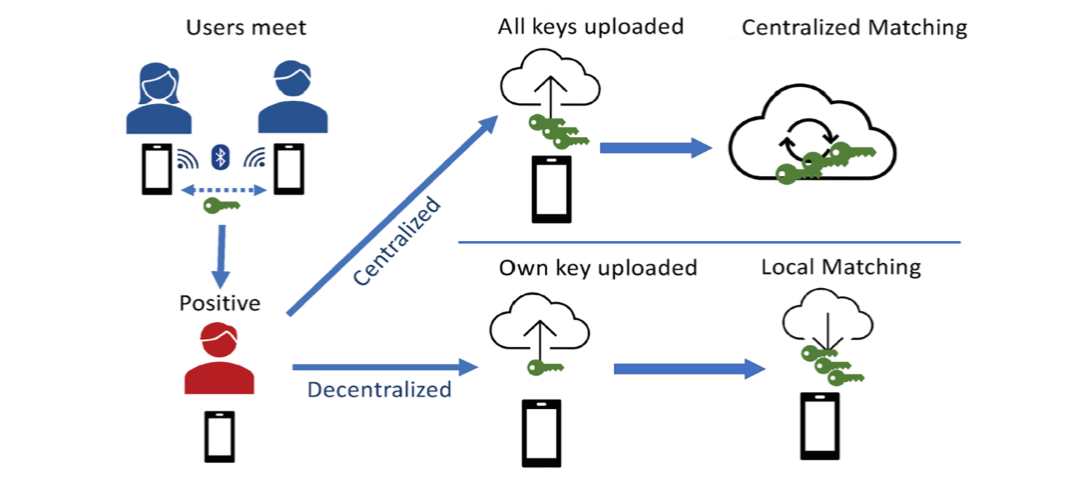
Centralized versus decentralized digital contact tracing. Reprinted from Hernández-Orallo et al [192] under the Creative Commons CC-BY 4.0 license.

The IDCTA must enable users to exchange and record temporary contact numbers when they are in contact within prespecified time and distance thresholds [44,154]. Temporary contact numbers should be renewed frequently (eg, every 15 minutes) to protect user privacy [44,141,154]. Contact logs of temporary contact numbers are maintained by each device, and once a case of COVID-19 is diagnosed, the DCTA allows them to notify their contacts [44,141]. How contact logs are processed to identify contacts and how contacts are alerted can be performed in a centralized or decentralized manner (Figure 2). Early in the pandemic, two predominant protocols emerged, the Pan-European Privacy Preserving Proximity Tracing (PEPP-PT initiative; centralized) and the Decentralized Privacy Preserving Proximity Tracing (DP-3T initiative; decentralized) [155,156]. With the PEPP-PT initiative, contact logs from the case’s device are processed centrally, while with the DP-3T initiative, contact logs are processed on the contact’s device by regularly checking a central server that holds the temporary contact numbers of cases [155,156]. The DP-3T initiative provides more protection to individual privacy and may enhance DCTA uptake [155,157]. The PEPP-PT initiative involves a human-in-the-loop, which is disadvantageous because it shares the case’s contact log with another individual. However, this may minimize false positives related to contact occurring through apartment walls or where adequate contact precautions were in place [156]. Centralized collection of personal data leaves individuals vulnerable to social network mapping and potentially having their movements mapped. Decentralized protocols may also be vulnerable to malicious attacks [133]. A user’s temporary contact number could be accessed and used by multiple devices and result in false contact chains being generated during the life cycle of that temporary contact number.

Ensuring processes that protect personal data from misuse are rigorously enforced is important in building public confidence that their personal data are safe. The European Union has stated that a DCTA should use a decentralized model to protect individual privacy [42]. They also emphasized that DCTAs should augment and not replace existing contact tracing systems [42]. Automated contact alert notification using a decentralized protocol may be alarming for contacts to receive. Therefore, the IDCTA should not only follow a decentralized privacy preserving protocol but also provide explicit instruction on what actions to take if a contact alert is received and a means of making contact with a health care worker for integration into the test and trace system.

How a contact is defined by the app should also be considered. DCTAs may define contacts according to binary distance (eg, within 2 m) and duration of contact (eg, 15 minutes or less) thresholds in keeping with the definition applied by health authorities [8,158,159]; although, this binary definition may not identify all contacts at risk of infection [160]. Alternatively, a risk-stratified approach that includes other factors associated with disease transmission may identify contacts more accurately [34]. Contact could be stratified as *high risk* or *low risk* depending on a risk score, with high-risk contacts being advised to quarantine and arrange COVID-19 testing and low-risk contacts being advised on good social distancing practice. Risk scores could be based on how close the contact was, for how long the contact lasted, how long it has been since the user met a COVID-19–positive person, and the risk of transmission for the case [161]. The risk of transmission would be based on empirical evidence of COVID-19 transmission dynamics. Temporary contact numbers recorded in the case’s DCTA each day would be assigned an additional code that represents the risk value of COVID-19 transmission from the case on that day. The contact’s DCTA would, in addition to recording the temporary contact number, record this additional code so that it may calculate the COVID-19 risk score when it matches temporary contact numbers from its contact log with that of a case. Risk scores are only as accurate as the data they are constructed from, and defining the risk score threshold would require an ongoing process of evaluation and calibration [42]. To calibrate the DCTA risk score effectively, knowledge of the contact event outcomes would be needed. To do this, the IDCTA would allow users to voluntarily have their COVID-19 test result uploaded directly to their DCTA from the processing laboratory (as is possible with the Corona-Warn app [115]) or for users to voluntarily confirm their status as having COVID-19 on the DCTA (as occurs with COVID Tracker [29] and COCOA [134]). The DCTA would have to centrally collect for users who receive a contact alert and who volunteer their COVID-19 test result, how long ago the contact occurred, the duration of contact, and the proximity of contact.

### Clinical and Societal Considerations

COVID-19 transmission chain disruption could potentially be enhanced by using DCTAs to augment manual contact tracing [16]. Therefore, this is the aim of the IDCTA. Other aims may be to act as a confidence-enhancing measure for those most vulnerable to COVID-19 infection. It may reassure them that they have not been a contact, and others can demonstrate their contact-free status to them [162]. The higher the uptake of the IDCTA, the more likely it is a case can notify a contact of their exposure. To achieve this, demonstrating to the target population the high degree of privacy preservation and adherence to data protection regulation is important. Enhancing uptake through small monetary incentives may also be considered [163,164]. The IDCTA should be voluntary [40,51-55,134], and mandating the use of DCTAs of uncertain effectiveness with associated potential harms, which have the highest likelihood of utility during periods of low COVID-19 incidence, may be difficult to justify as a proportionate response.

The IDCTA should avoid functions that necessitate additional data processing that may raise privacy concerns, such as age, sex, location, or ethnicity. Any additional functions should be justifiable, proportionate, privacy preserving, and adherent to data protection regulation. Additional functions should be defined before DCTA deployment in keeping with the data protection principle of purpose limitation [128]. DCTAs present an opportunity to perform functions such as allowing people to assess their personal risk of being hospitalized or dying from COVID-19. Providing risk assessments may not be ethical given that risk algorithms may be population specific, not generalizable, and may provide falsely elevated or falsely lowered risks [165]. Regardless of risk, it could be argued that the person’s behavior should be the same and an awareness of one’s risk may not result in positive behavior change. Therefore, the IDCTA would not provide risk assessments. Symptom checker functions should also be avoided because there is little high-quality evidence to support their use in this context.

### Evaluation Considerations

To be ethical and adherent with data protection regulation, the continued use of a DCTA needs to be supported by evidence that it has been effective in contributing to epidemic control. A DCTA is a multistep intervention. There are several steps where they may fail to effectively disrupt transmission chains, including being downloaded; recording contact events; sending contact alerts; and integrating with the wider contact tracing, testing, quarantine, and isolation systems [16,166]. Maintaining privacy while ensuring the necessary data to demonstrate effectiveness are collected is challenging. To enable evaluation of effectiveness, the IDCTA should record and collect key metrics (Table 2). These were derived from ECDC guidance on how to monitor contact tracing effectiveness [159], the limited number of studies evaluating real-world DCTA effectiveness [150,151,167-170], and other published academic literature [62]. Many of these metrics may be collected by the app and do not interfere with individual privacy. Other metrics such as the outcome of COVID-19 testing would be considered sensitive data by many. Collection of these sensitive data should be voluntary. Determining whether people who receive contact alerts quarantine, a key intervention in disrupting transmission changes, may be difficult. This is true for both digital and manual contact tracing. GPS location tracking has been used in China by public health authorities to confirm contacts remain within quarantine [171]. However, this interference with the right to privacy would likely not be acceptable in countries where privacy is highly valued. The outcomes of the evaluation should be openly reported to allow for a wider public evaluation of the app.

**Table 2:** Metrics to evaluate ideal DCTA effectiveness.

Indicator of effectiveness	Purpose	Metric numerator (source)	Metric denominator (source)
DCTA^a^ is downloaded	To estimate the proportion of the smartphone owning population who download the DCTA	Number of DCTA downloads minus number of DCTA deletions (DCTA)	Number of smartphone owners nationally (Government statistics office; eg, Central Statistics Office, ROI^b^)
DCTA is active	To estimate the proportion of DCTAs downloaded that are being used	Number of DCTAs with contact tracing turned on (DCTA)	Number of DCTAs downloaded minus number of DCTAs deleted (DCTA)
DCTA is active	To estimate the proportion of DCTAs downloaded that are being used	Frequency and duration of use (DCTA)	N/A^c^
DCTA is active	To estimate the proportion of DCTAs downloaded that are being used	Number of DCTAs downloading TCNs^d^ of cases on central server per day (assuming DCTA downloads keys once per day when active; DCTA)	Number of DCTAs downloaded minus number of DCTAs deleted (DCTA)
DCTA is used by COVID-19 cases	To estimate the DCTA penetration among people who contract COVID-19	Number of positive test results uploaded to DCTA (DCTA)	Number of COVID-19 cases nationally (national surveillance data)
DCTA is used by COVID-19 cases	To estimate the DCTA penetration among people who contract COVID-19	Number of COVID-19 cases who attended a screening center reporting DCTA active use (survey of attendees at testing centers and review of participants’ test results)	Number of COVID-19 cases who attended a screening center (screening center data)
DCTA is used by COVID-19 cases to notify close contacts	To estimate the proportion of cases using the DCTA who use it to send contact alerts	Number of DCTAs that send a contact alert (DCTA)	Number of DCTAs with a positive COVID-19 test recorded (national surveillance data)
DCTA is used by COVID-19 cases to notify close contacts	To estimate the proportion of cases using the DCTA who use it to send contact alerts	Number of COVID-19 cases who attended a screening center reporting DCTA active use and who report sending a contact alert (follow-up survey of COVID-19 cases who reported DCTA use at time of screening)	Number of COVID-19 cases who attended a screening center reporting DCTA active use (survey of attendees at testing centers and review of participants’ test results)
Close contacts using DCTA receive alert	To estimate the DCTA penetration among people who are close contacts	Number of DCTAs that receive a contact alert (DCTA)	Number of close contacts identified nationally (national surveillance data)
DCTA identifies contacts not identified by manual contact tracing	To demonstrate the DCTA augments manual contact tracing	Number of close contacts attending testing center identified exclusively by DCTA (survey of attendees at testing centers)	Number of close contacts attending testing center (survey of attendees at testing centers)
DCTA identifies contacts sooner than manual contact tracing	To demonstrate the DCTA augments manual contact tracing	Number of close contacts attending testing center who received contact alert from DCTA before contact alert from manual contact tracing service (survey of attendees at testing centers)	Number of close contacts attending testing center (survey of attendees at testing centers)
Close contacts using DCTA are tested for COVID-19	To estimate the proportion of contacts who are tested for COVID-19 and to estimate the number of cases identified by the DCTA	Number of DCTAs with a COVID-19 test result uploaded within 14 days of a contact alert (DCTA)	Number of DCTAs that receive a contact alert (DCTA)
DCTA associated harm is recognized	To determine what harms, if any, occur with DCTA use	N/A (qualitative survey of DCTA users)	N/A

^a^DCTA: digital contact tracing app.

^b^ROI: Republic of Ireland.

^c^N/A: not applicable.

^d^TCN: temporary contact number.

### Summary of Findings

Key considerations were ethical, user experience, privacy and data protection, clinical and societal, and evaluation. Proportionality, voluntariness, transparency, trustworthiness, and equity are necessary for the design and deployment of the IDCTA. Universality and user engagement are important user experience considerations that can influence DCTA use in the population. Dimensions of universality that should be taken into account when designing the IDCTA are accessibility, minors as users, cultural universality, content, availability, and maintenance and frequency of upgrades. User engagement could be enhanced by small financial incentives, enabling users to tailor aspects of the app to their particular needs and integrating DCTAs into the wider public health information campaign. If DCTAs are to be trusted, accepted, and used by the target population, they must be adherent to data protection regulation and have privacy by design through all elements, including maintaining contact logs, generating contact alerts, and linking users into the test and trace system. For the IDCTA, the choice of which technology is used will be influenced by its cost, energy use, availability, accessibility, adherence to data protection regulation and principles of privacy by design, and accuracy when detecting contact events. Combining ultrasound technology with Bluetooth LE may improve accuracy by reducing the number of false-positive contacts identified. A decentralized privacy preserving protocol should be followed to enable DCTA users to exchange and record temporary contact numbers during contact events. The IDCTA should define and risk stratify contact events according to proximity, duration of contact, and the infectiousness of the case at the time of contact. Evaluating DCTAs requires data to quantify app downloads, use among COVID-19 cases, successful contact alert generation, contact alert receivers, contact alert receivers that adhere to quarantine and testing recommendations, and the number of contact alert receivers who subsequently are tested positive for COVID-19.

## Discussion

### Principal Findings

This cross-disciplinary review presents best practice guidance for developing the IDCTA and is informative for those involved in DCTA research, design, and deployment. It also serves as a comprehensive and accessible entry point for those beginning to engage with this research subject, which has evolved significantly after a period of intensive exploration in 2020. DCTAs will likely be a significant research field not only for the remainder of the COVID-19 pandemic but also in the postpandemic era because of a renewed interest and support for pandemic preparedness. Demonstrating the effectiveness of COVID-19 DCTAs is a current research priority [193]. This is important to convince not only nonapp users of their benefits but also current or previous app users, many of whom remain uncertain about their utility [194]. Early evidence indicates that DCTAs can identify contacts of COVID-19 cases who subsequently develop infection (particularly among nonhousehold contacts) [150,151,167-170], may shorten the time to quarantine by 1 day [168], and can prevent further disease transmission [169]. Ensuring DCTAs are integrated with the wider test and trace system is emerging as an important aspect of DCTA deployment [135]. Where codes were required by DCTA users to confirm on the app a COVID-19–infected status, manual distribution of these codes by health care professionals could delay contact alert generation and subsequent downstream actions such as contact quarantine and testing, suggesting automated code generation is preferable [135].

A weakness of this research was that it did not specifically address how DCTAs should be integrated into the wider test and trace system. There is a need for future dedicated research to synthesize and evaluate evidence, and generate best practice recommendations for this consideration of DCTA deployment. The limitations of this review are that the index and gray literature searches, while extensive, were not performed using systematic review methodology. The inclusion of both indexed and gray literature enabled the derivation of best practice guidance from the literature during a phase of rapid DCTA research and development growth. The cross-disciplinary approach taken to evaluating the evidence was a strength of this research because it allowed varying aspects of DCTA design and deployment to be considered.

Future promising developments in this field may be the use of blockchain technology, ultra-wideband technology, and artificial intelligence in DCTA design. Privacy and data protection concerns are significant barriers to DCTA uptake in Western societies [72,74-76,122-124]. A blockchain network is a decentralized, distributed, and secure public ledger that stores records of transactions securely using cryptography techniques [195]. Features of blockchain technology that make it advantageous for digital contact tracing are decentralized data storage; data security through encryption; data provenance and time stamping allowing for verification of the data legitimacy and data immutability, which enhances data reliability and transparency [196]. The use of blockchain networks in future DCTAs may reduce privacy and data protection concerns and enhance DCTA uptake and use [196]. This area should be a focus of future research. DCTAs need to detect contact events accurately to optimize uptake [79,105,144,145]. Ultra-wideband is a low energy means of enabling short-range high bandwidth communications that can transmit data with minimal noise interference. This could allow for highly accurate measurement of contact events within centimeters [197]. Although not yet a feature of most smartphones, it most likely will be in the near future [198]. Therefore, it could be a viably accessible and available energy efficient technology for DCTAs in future pandemics. Additionally, artificial intelligence could potentially improve the accuracy of future DCTA contact event detection by reducing false positives and false negatives [199].

### Conclusion

In conclusion, key considerations and best practice guidance for the design of the IDCTA were derived from the literature.

## Appendix

Multimedia Appendix 1 Cross-disciplinary approach.

Multimedia Appendix 2 Scoping review.

Multimedia Appendix 3 Search strategy.

Multimedia Appendix 4 Gray literature sources.

Multimedia Appendix 5 Included articles' description.

Multimedia Appendix 6 Ethical frameworks for digital contact tracing apps.

Multimedia Appendix 7 Evidence supporting user experience recommendations.

## Abbreviations

API: application programming interface
DCTA: digital contact tracing app
DP-3T: Decentralized Privacy Preserving Proximity Tracing
ECDC: European Centre for Disease Prevention and Control
EDPB: European Data Protection Board
GDPR: General Data Protection Regulation
IDCTA: ideal digital contact tracing app
LE: Low Energy
NHS: National Health Service
PEPP-PT: Pan-European Privacy Preserving Proximity Tracing
ROI: Republic of Ireland
WHO: World Health Organization
