# Supplementary material for: Best Practice Guidance for Digital Contact Tracing Apps: A Cross-disciplinary Review of the Literature
Source: JMIR Mhealth Uhealth. 2021 Jun 7;9(6):e27753. doi: 10.2196/27753 (PMC8189288; doi:10.2196/27753)
Supplement: Multimedia Appendix 1 [file mhealth_v9i6e27753_app1.docx]

Appendix 1: Cross Disciplinary Approach

COVIGILANT was a Science Foundation Ireland funded cross disciplinary research project led by Lero, the Software Research Centre at the University of Limerick [1]. COVIGILANT initiated its work on 27^th^ of May 2020 [1]. The aim of the project was to optimize digital contact tracing from an end user, a current practice, and an idealized solution perspective. There were 21 members of the cross disciplinary team (who are the authors of this article). Team members were general practitioners, intensive care medicine specialists, hospital specialists, medical technologists and mhealth specialists, engineers, statisticians, contact tracers and computer science and information system specialists with subspecialties in security, software evaluation and usability. Team members worked within one or more of three research streams as part of a cross disciplinary approach (Figure 1) to achieving the aim of the COVIGILANT research project:

Stream 1 aimed to identify end-users’ perceptions of digital contact tracing via large-scale public surveys, coupled with focus-group studies [4].

Stream 2 aimed at developing a compare-and-contrast framework for analysis of existing contact tracing applications, and using it to review these applications, to identify best-of-breed practice [2,3,5].

Stream 3 aimed to derive and summarize best practice guidance on the design of an idealized digital contact tracing app.


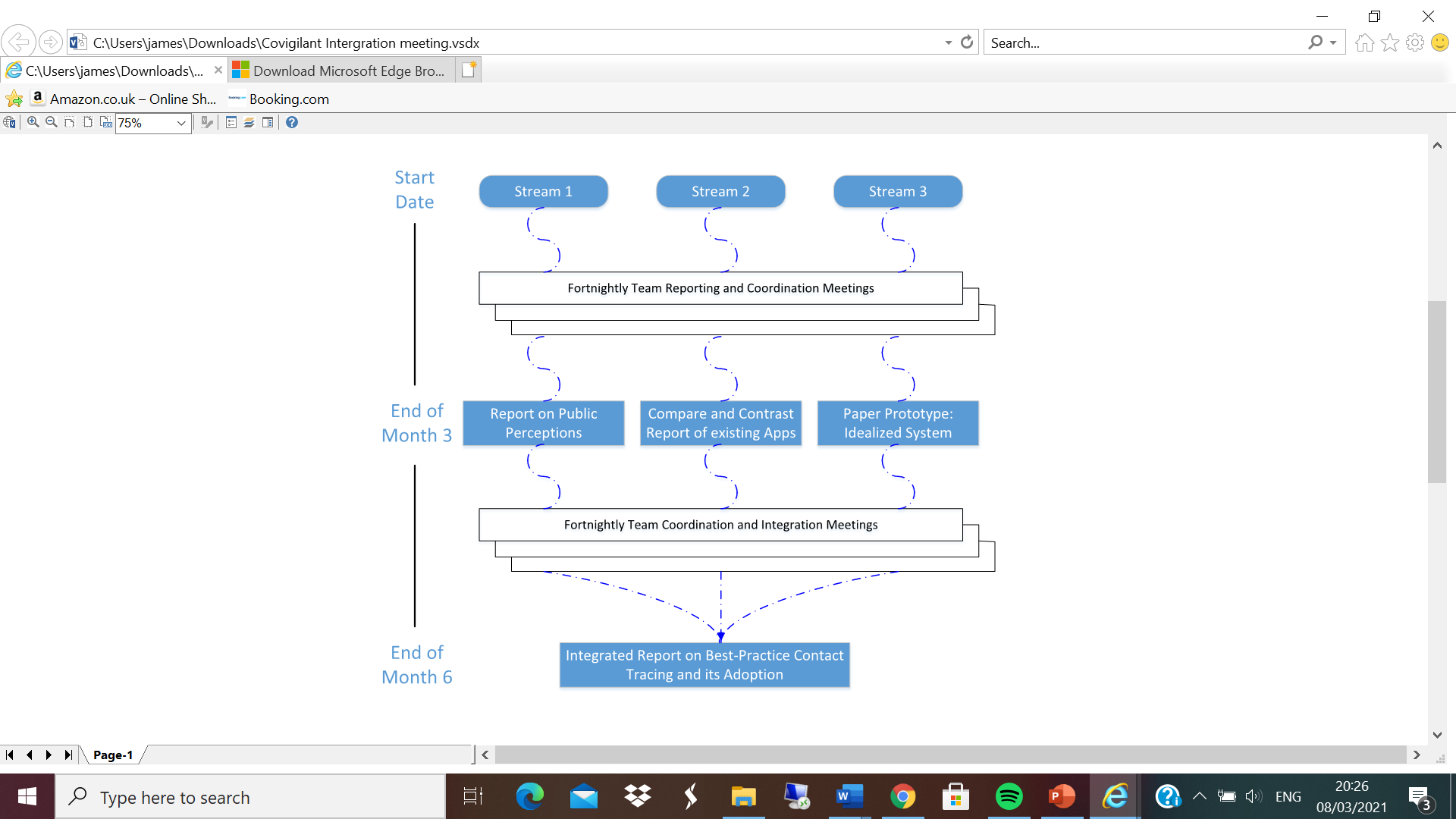


Figure 1 COVIGILANT cross disciplinary approach

Author 1 performed the search of the indexed (Appendix 3) and grey literature (Appendix 4), focusing on one of the six key considerations (identified from the scoping review described in Appendix 2) and reporting on that at bi-weekly intervals (Table 1, work-packages 3.1, 3.2, 3.3, 3.4). For every work-package, a report describing the evidence and considerations described in the literature was constructed by author 1 and refined through a review and feedback cycle involving all members of stream 3. The cycle of review and feedback was repeated until there was cross-disciplinary agreement that all feedback had been adequately addressed. Following this, the findings in these reports were synthesized into one report and refined through similar discussion with streams 1 and 2 (Table 1 work-packages 3.5, 3.6, 3.7). The final report (this article) was drafted after considering and integrating with the final research outputs from streams 1 and 2, many of which have been published in peer-reviewed venues [2,3][4][5].

Table 1: Stream 3 Work-packages

| Work-package | Deliverable | Delivery Date |
| --- | --- | --- |
| 3.1: Perspectives identification. | Report detailing the multiple aspects (clinical and societal, technical and user experience, privacy, and data protection and ethical) that need to be considered in an IDTCA. | July  15^th^ 2020 |
| 3.2: Clinical and societal considerations evaluation. | Report outlining the Clinical Issues. | August 1^st^ 2020 |
| 3.3: Technical & User experience considerations evaluation. | Report outlining the Technical & UX issues. | August  15^th^ 2020 |
| 3.4: Privacy, data protection and ethical considerations evaluation. | Report outlining the Societal (e.g. privacy, security and regulation) issues. | September 1^st^ 2020 |
| 3.5: Presentation of results. | Summary report on IDCTA. | October  1^st^ 2020 |
| 3.6: Reflection on, and refinement of, the individual considerations. | Updated reports on idealized clinical and societal, technical and user experience, and privacy and data protection and ethical considerations. | November 1^st^ 2020 |
| 3.7: Updated results presentation. | Report outlining updated summary findings in the light of contributions from streams 1 and 3. | December 31^st^ 2020 |

References

1. Buckley J et al. Covigilant: Optimizing Digital Contact Tracing from End-User/Current Practice/Idealized-Solution perspectives. A Proposal for the SFI Covid-19 Rapid Response Funding Call Lero Technical Report: 2020-TR-05 https://lero.ie/sites/default/files/2020-TR-05_Covigilant%20SFI%20Application%20Tech%20Report.pdf.

2. Rekanar K, O’Keeffe IR, Buckley S, Abbas M, Beecham S, Chochlov M, et al. Sentiment analysis of user feedback on the HSE’s Covid-19 contact tracing app. Irish Journal of Medical Science (1971-). Springer; 2021;1–10.

3. Storni C, Tsvyatkova D, Richardson I, Buckley J, Abbas M, Beecham S, et al. Toward a Compare and Contrast Framework for COVID-19 Contact Tracing Mobile Applications: A Look at Usability. 2021;

4. O’Callaghan ME, Buckley J, Fitzgerald B, Johnson K, Laffey J, McNicholas B, et al. A national survey of attitudes to COVID-19 digital contact tracing in the Republic of Ireland. Irish Journal of Medical Science (1971-). Springer; 2020;1–25.

5. Welsh T, Rekanar K, Abbas M, Chochlov M, Fitzgerald B, Glynn L, et al. Towards a taxonomy for evaluating societal concerns of contact tracing apps. 2020 7th International Conference on Behavioural and Social Computing (BESC). 2020. p. 1–6.
