# Supplementary material for: Best Practice Guidance for Digital Contact Tracing Apps: A Cross-disciplinary Review of the Literature
Source: JMIR Mhealth Uhealth. 2021 Jun 7;9(6):e27753. doi: 10.2196/27753 (PMC8189288; doi:10.2196/27753)
Supplement: Multimedia Appendix 2 [file mhealth_v9i6e27753_app2.docx]

Appendix 2: Scoping Review

On inception of the COVIGILANT research project (Appendix 1) [1] in May 2020 the research team reviewed the available guidance from the World Health Organisation [2], European Centre for Disease Control [3], European Data Protection Board [4], European Union eHealth Network [5] and TraceTogether [6], a white paper report from the Ada-Lovelace institute [7] and a peer reviewed publication describing a modelling study of the potential effect of digital contact tracing on COVID-19 outbreak control [8]. From these, the following considerations were identified by the COVIGILANT team members:

1) Ethical

2) User experience

3) Privacy

4) Data protection

5) Technical

6) Clinical

7) Societal

After Stream 3 completed work-package 3.1 (perspectives identification) these were refined, after discussion with members of Streams 1 and 2, to re-group together considerations which the team considered being strongly interrelated. Specifically, data protection and privacy were clustered together and clinical/societal considerations were clustered together based on their logical coherence as privacy and domain considerations respectively:

1) Ethical considerations

2) User experience considerations

3) Privacy and data protection considerations

4) Technical considerations

5) Clinical and societal considerations

After completion of work-package 3.5 Summary Report on the ideal digital contact tracing app, that was shared with and discussed between the members of the three streams of the project, it became evident that many of the issues that arose in work-packages 3.1-3.5 were dependent on how digital contact tracing apps would be evaluated for effectiveness and if evidence supporting their effectiveness was demonstrable. Therefore, a sixth consideration “evaluation” was added. This resulted in the definitive list of considerations, as described in this article, being:

1. Ethical considerations

2. User experience considerations

3. Privacy and data protection considerations

4. Technical considerations

5. Clinical and societal considerations

6. Evaluation considerations

References

1. J Buckley et al. Covigilant: Optimizing Digital Contact Tracing from End-User/Current Practice/Idealized-Solution perspectives. A Proposal for the SFI Covid-19 Rapid Response Funding Call Lero Technical Report: 2020-TR-05 https://lero.ie/sites/default/files/2020-TR-05_Covigilant%20SFI%20Application%20Tech%20Report.pdf.

2. World Health Organisation. Contact tracing in the context of COVID-19: interim guidance, 10 May 2020. 2020;

3. European Centre for Disease Control. Mobile applications in support of contact tracing for COVID-19, 2020. [cited 2021 Feb 4]; Available from: https://www.ecdc.europa.eu/sites/default/files/documents/covid-19-mobile-applications-contact-tracing.pdf

4. Guidelines on the use of location data and contact tracing tools in the context of the COVID-19 outbreak. European Data Protection Board, 2020 .

5. Mobile Applications to support contact tracing in the EU’s fight against COVID-19. Common EU Toolbox for Member States,European eHealth Network, 2020;1-56.

6. Bay J, Kek J, Tan A, Hau CS, Yongquan L, Tan J, et al. BlueTrace: A privacy-preserving protocol for community-driven contact tracing across borders. Government Technology Agency-Singapore, Tech Rep. 2020;

7. Ada Lovelace institute. Exit through the App Store? Rapid Evidence Review, April 2020. [cited 2021 Feb 4]; Available from: https://www.adalovelaceinstitute.org/wp-content/uploads/2020/04/Ada-Lovelace-Institute-Exit-through-the-App-Store-Explainer-for-Government-April-2020.pdf

8. Ferretti L, Wymant C, Kendall M, Zhao L, Nurtay A, Abeler-Dӧrner L, et al. Quantifying SARS-CoV-2 transmission suggests epidemic control with digital contact tracing. Science. American Association for the Advancement of Science; 2020;368(6491).
