# Supplementary material for: Best Practice Guidance for Digital Contact Tracing Apps: A Cross-disciplinary Review of the Literature
Source: JMIR Mhealth Uhealth. 2021 Jun 7;9(6):e27753. doi: 10.2196/27753 (PMC8189288; doi:10.2196/27753)
Supplement: Multimedia Appendix 3 [file mhealth_v9i6e27753_app3.docx]

Appendix 3: Search Strategy

Ovid Medline (R) and Epub Ahead of Print, In-Process & Other Non-Indexed Citations, Daily and Versions(R) search performed 3/7/20 and updated monthly, most recently 10/03/21.

1. (COVID* or SARS CoV 2 or novel coronavirus or nCoV 2019 or SARS or SARS CoV or pandemic* or coronavirus* or sars cov 2 or sars coronavirus 2 or novel coronavirus or novel corona virus or coronavirus infection*).ab,ti.

2. (Smartphone application* or smartphone* or smart phone* or cell phone* or cellular phone* or mobile phone* or digital solution* or GPS device* or GPS track* or ICTs or Bluetooth or health apps or health application* or eHealth or mHealth or mobile app* or digital technolog* or digital health or smart watch* or telemedicine or digital contact tracing).ab,ti.

3. (contact adj3 (trac* or examination or screening or management or investigation)).ab,ti.

4. exp Coronavirus/ or exp Coronavirus Infections/

5. exp Contact Tracing/

6. exp Mobile Applications/

7. exp Smartphone/

8. 1 or 4

9. 2 or 6 or 7

10. 3 or 5

11. 8 and 9 and 10
