# Supplementary material for: Best Practice Guidance for Digital Contact Tracing Apps: A Cross-disciplinary Review of the Literature
Source: JMIR Mhealth Uhealth. 2021 Jun 7;9(6):e27753. doi: 10.2196/27753 (PMC8189288; doi:10.2196/27753)
Supplement: Multimedia Appendix 4 [file mhealth_v9i6e27753_app4.docx]

Appendix 4: Grey Literature Sources

| Organisation | Site |
| --- | --- |
| Public Health Authorities | |
| World Health Organisation Institutional Repository for Information Sharing | https://apps.who.int/iris/ |
| European Centre for Disease Control | <https://www.ecdc.europa.eu/en> |
| Centre for Disease Control | [www.cdc.gov](http://www.cdc.gov) |
| Africa Centre for Disease Control and Prevention | https://africacdc.org/ |
| Health Protection Surveillance Centre (Ireland) | [www.hpsc.ie](http://www.hpsc.ie) |
| Public Health Agency (Northern Ireland) | https://www.publichealth.hscni.net/ |
| Public Health Scotland | https://publichealthscotland.scot/ |
| Public Health Wales | https://phw.nhs.wales/ |
| Public Health England | <https://www.gov.uk/government/organisations/public-health-england> |
| Austrian National Public Health Institute (Gesundheit Österreich GmbH, GÖG) | https://goeg.at/ |
| Danish Health Authority | <https://www.sst.dk/da> |
| Federal Office of Public Health (Switzerland) | https://www.bag.admin.ch/bag/de/home.html |
| Finnish Institute for Health and Welfare | <https://thl.fi/en/web/thlfi-en> |
| Carlos III Health Institute (Spain) | <https://eng.isciii.es/eng.isciii.es/Paginas/Inicio.html> |
| National Institute of Health (Italy) | https://www.iss.it/ |
| National Institute of Public Health – National Institute of Hygiene (Poland) | https://www.pzh.gov.pl/ |
| National Public Health Organization (Greece) | http://eody.gov.gr/ |
| RIVM (Netherlands) | <https://www.rivm.nl/en> |
| Norwegian Institute of Public Health (NIPH) | https://www.fhi.no/ |
| Public Health Agency of Sweden | https://www.folkhalsomyndigheten.se/the-public-health-agency-of-sweden/ |
| Robert Koch Institute (Germany) | [www.rki.de](http://www.rki.de) |
| Santé publique France (France) | https://www.santepubliquefrance.fr/ |
| Public Health Agency of Canada | https://www.phac-aspc.gc.ca/ |
| National Institute for Communicable Disease (South Africa) | https://www.nicd.ac.za/ |
| Korea Disease Control and Prevention Agency | https://www.cdc.go.kr/cdc_eng/ |
| National Centre for Disease Control (India) | https://ncdc.gov.in/ |
| National Centre for Infectious Diseases (Singapore) | https://www.ncid.sg/Pages/default.aspx |
| National Institute of Public Health of Japan (Japan) | <https://www.niph.go.jp/index_en.html> |
| Ministry of Health (New Zealand) | https://www.health.govt.nz/ |
| Department of Health (Australia) | https://www.health.gov.au/ |
| Ministry of Public Health (Qatar) | https://www.moph.gov.qa/english/Pages/default.aspx |
| Ministry of Health (Israel) | <https://www.health.gov.il/English/Pages/HomePage.aspx> |
| Digital Contact Tracing Apps | |
| Stop Corona App (Austria) | <https://www.austria.info/en/service-and-facts/coronavirus-information/app> |
| Coronalert (Belgium) | https://coronalert.be/en/ |
| Smittestop (Denmark) | https://smittestop.dk/ |
| Koronavilkku (Finland) | https://koronavilkku.fi/ |
| TousAntiCovid (France) | <https://www.economie.gouv.fr/tousanticovid> |
| Corona-Warn-App (Germany) | <https://www.bundesregierung.de/breg-de/themen/corona-warn-app/corona-warn-app-englisch> |
| COVID-Tracker (Ireland) | https://covidtracker.gov.ie/ |
| Smittestopp (Norway) | https://www.helsenorge.no/en/smittestopp/ |
| CoronaMelder (Netherlands) | https://coronamelder.nl/en/ |
| Stop COVID (Poland) | <https://www.gov.pl/web/protegosafe> |
| Radar COVID (Spain) | https://radarcovid.gob.es/ |
| TraceTogether (Singapore) | https://www.tracetogether.gov.sg/ |
| COVID Safe (Australia) | <https://www.health.gov.au/resources/apps-and-tools/covidsafe-app> |
| NHS COVID-19 App (UK) | https://www.covid19.nhs.uk/ |
| PathCheck/SafePaths (MIT, USA) | https://www.pathcheck.org/ |
| Ehteraz (Qatar) | <https://portal.moi.gov.qa/wps/portal/ar> |
| NOVID (USA) | NOVID.org |
| COVID Tracer (New Zealand) | https://tracing.covid19.govt.nz/ |
| Swiss COVID (Switzerland) | <https://www.bag.admin.ch/bag/de/home.html> |
| Aarogya Setu (India) | https://www.aarogyasetu.gov.in/ |
| Aman (Jordan) | <https://amanapp.jo/en> |
| Media | |
| New York Times | https://www.nytimes.com/ |
| Raidió Teilifís Éireann News | Rte.ie |
| British Broadcasting Corporation News | bbc.com/news |
| Wall Street Journal | https://www.wsj.com/ |
| Financial Times | [www.ft.com](http://www.ft.com) |
| The Guardian | <https://www.theguardian.com/international> |
| Harvard Business Review | https://hbr.org/ |
| MIT Technology Review | https://www.technologyreview.com/ |
| Reuters | Reuters.com |
| Irish Times | Irishtimes.com |
| British Medical Journal News | <https://www.bmj.com/news> |
| Nature (News and Comments) | <https://www.nature.com/news> |
| Silicon Republic | https://www.siliconrepublic.com/ |
| Other | |
| Our World in Data | https://ourworldindata.org/ |
| Blog Technology in the NHS | https://healthtech.blog.gov.uk/ |
| Norton Rose Fulbright | <https://www.nortonrosefulbright.com/en-il> |
| Bluetrace | https://bluetrace.io/ |
| Pan-European Privacy-Preserving Proximity Tracing | <https://github.com/pepp-pt> |
| Decentralized Privacy-Preserving Proximity Tracing | <https://github.com/DP-3T> |
| Ada Lovelace Institute | https://www.adalovelaceinstitute.org/ |
| COVID-19 Digital Rights Tracker | https://www.top10vpn.com/research/investigations/covid-19-digital-rights-tracker/ |
| Amnesty International | Amnesty.org |
| United Nations Human Rights Office of the High Commissioner | <https://www.ohchr.org/EN/Pages/Home.aspx> |
| Cochrane | Cochrane.org |
| Centre for Evidence-Based Medicine | https://www.cebm.net/ |
| eHealth Network | https://ec.europa.eu/health/ehealth/policy/network_en |
| Xcertia | <https://www.mobihealthnews.com/tag/xcertia> |
| European Data Protection Board | <https://edpb.europa.eu/edpb_en> |
| Data Protection Commission | https://www.dataprotection.ie/ |
| European Telecommunication Standards Institute | https://www.etsi.org/ |
| European Commission | https://ec.europa.eu/info/live-work-travel-eu/coronavirus-response_en |
| Nuffield Council on Bioethics | https://www.nuffieldbioethics.org/ |
| arXiv preprint server | https://arxiv.org/ |
| JMIR preprint server | https://preprints.jmir.org/ |
| MedRxiv preprint server | https://www.medrxiv.org/ |
