# Supplementary material for: Best Practice Guidance for Digital Contact Tracing Apps: A Cross-disciplinary Review of the Literature
Source: JMIR Mhealth Uhealth. 2021 Jun 7;9(6):e27753. doi: 10.2196/27753 (PMC8189288; doi:10.2196/27753)
Supplement: Multimedia Appendix 5 [file mhealth_v9i6e27753_app5.docx]

Appendix 5: Included Articles

|  | Author (Year) | Country (of study/  authors) | Publication type | Description | Consideration | Source |
| --- | --- | --- | --- | --- | --- | --- |
| 1 | Scottish Human Rights Commission (2020) [1] | Scotland | Briefing report | Describes the human rights implications of digital contact tracing. | Ethical | Grey literature |
| 2 | Ada Lovelace Institute (2020) [2] | United Kingdom | Review | Review of technological interventions to combat COVID-19 | Ethical, Privacy and data protection, Technical | Grey literature |
| 3 | Ferretti et al. (2020)[3] | United Kingdom | Original research | Modelling of the potential impact of digital contact tracing on COVID-19 epidemics. | Ethical, Privacy and data protection, Technical | Indexed literature search |
| 4 | Nature [4] | N/a | Perspective | Discussion of need for digital contact tracing apps to be shown to be effective and secure. | Privacy and data protection | Grey literature |
| 5 | Upshur R. (2002) [5] | Canada | Conceptual analysis and literature review | Discusses principles relevant to ethical deliberation in public health. | Ethical | References of included articles |
| 6 | Childress RF et al. (2012) [6] | United States of America | Perspective | Provide a conceptual map of the terrain of public health ethics. | Ethical | References of included articles |
| 7 | Kass N et al. (2001) [7] | United States of America | Perspective | A description of a six-step ethical framework devised to help public health professionals analyze the ethical implications of proposed interventions. | Ethical | References of included articles |
| 8 | Parker MJ et al. (2020) [8] | United Kingdom | Perspective | Discussion of the ethical considerations relating to digital contact tracing and COVID-19. | Ethical, Privacy and data protection | References of included articles |
| 9 | World Health Organization (2020) [9] | N/a | WHO Guidance | Ethical considerations to guide the use of digital proximity tracking technologies for COVID-19 contact tracing | Ethical, Privacy and data protection | Grey literature |
| 10 | World Health Organisation (2020) [10] | N/a | WHO Guidance | Interim guidance on contract tracing | Technical | Grey literature |
| 11 | Ranisch R. et al. (2020) [11] | Sweden, Germany, Singapore | Perspective | Describes an ethical framework for the design and implementation of digital contact tracing. | Ethical, Privacy and Data protection | Indexed literature search |
| 12 | Lockey S. et al (2021) [12] | Australia | Original research | Survey profiling adopters and non-adopters of contact tracing applications | Ethical | Indexed literature search |
| 13 | Gasser et al. [13] | United States of America, Switzerland | Perspective | A typology of digital public health interventions for COVID-19 and a navigation aid for policy makers | Ethical, Privacy and data protection | Indexed literature search |
| 14 | Lo B. et al. (2020) [14] | United States of America | Perspective | An analysis of ethical issues in contact tracing and a framework for assessing contact tracing | Ethical, Privacy and data protection, Clinical and societal | Indexed literature search |
| 15. | Morley J. et al. (2020) [15] | United Kingdom | Perspective | Description of questions to answer when assessing whether contact tracing apps are ethical | Ethical, Privacy and data protection | Indexed literature search |
| 16 | European Commission [16] | European Union | Press release | Description of Union recommendation for a common toolbox for the use of technology to combat COVID-19 | Ethical | Grey literature |
| 17 | Nuffield Council on Bioethics (2020) [17] | United Kingdom | Briefing report | This rapid policy briefing outlines ethical principles that must be considered when developing public health measures in response to the COVID-19 pandemic | Ethical, Privacy and data protection | Grey literature |
| 18 | Sharon T. (2020) [18] | Netherlands | Perspective | Discussion of the role of technology companies in pandemic response. | Ethical, Privacy and data protection | Indexed literature search |
| 19 | Storeng K. et al. (2021) [19] | Norway | Perspective | Discussion of the relationship between technology companies and public health authorities in pandemic response | Ethical, Privacy and data protection | Indexed literature search |
| 20 | Williams S. et al. (2021) [20] | United Kingdom | Original research | Survey of UK residents about their attitudes to digital contact tracing | Ethical, Privacy and data protection | Indexed literature search |
| 21 | Braithwaite I. (2020) [21] | United Kingdom | Systematic review and meta-analysis | Assesses the effectiveness of automated and partly automated contact-tracing systems for COVID-19 | Ethical | References of included articles |
| 22 | Angelmeyer A. et al. (2020) [22] | Cochrane review group | Rapid review | Assesses the benefits and harms of digital technologies in identifying contacts of infectious disease carrier. | Ethical, Technical | Indexed literature search |
| 23 | Shamil S. et al. (2021) [23] | United States of America | Original research | Modelling study describing the effects of measures to contain COVID-19 epidemics including digital contact tracing | Ethical | Indexed literature search |
| 24 | von Wyl V. et al. (2020) [24] | Switzerland | Perspective | Discusses an agenda for evaluating digital proximity tracing apps. | Ethical, Evaluation | References of included articles |
| 25 | Roderick P. et al (2020) [25] | United Kingdom | Perspective | Discusses challenges and solutions for the UK contact tracing system. | Ethical | Grey literature |
| 26 | New York Times [26] | United States of America | Press release | Discusses challenges and solutions for the USA contact tracing system. | Ethical | Grey literature |
| 27 | Baumgärtner L et al. (2020) [27] | Germany | Perspective | Discussion of security and privacy risks with app based contact tracing and potential solutions. | Ethical | Preprint server |
| 28 | British Medical Journal (2020) [28] | United Kingdom | Press release | Describes high financial cost of information technology interventions in healthcare. | Ethical | Grey literature |
| 29 | von Wyl et al. (2021) [29] | Switzerland | Original research | Analysis of swiss media coverage of digital contact tracing app. | Ethical, Privacy and data protection | Indexed literature search |
| 30 | Berman G. et al. (2020) [30] | UNICEF | Working paper | Describes general and child-specific ethical issues relating to digital contact tracing. | Ethical, Privacy and data protection, Technical | Grey literature |
| 31 | Leith DJ (2020) [31] | Ireland | Original research | Measurement-based evaluation of Google/Apple Exposure Notification API for proximity detection in a light-rail tram | Ethical, Technical | Indexed literature search |
| 32 | European Centre for Disease Control [32] | Europe | ECDC Guidance | Outlines several epidemiological and operational issues that public health authorities and app developers should consider | Ethical, technical, privacy and data protection, clinical and societal | Grey literature |
| 33 | COVID Tracker [33] | Ireland | GitHub with app algorithms and documentation | Facilitates transparency by allowing public access to app algorithms and documentation | Ethical | Grey literature |
| 34 | Corona-Warn-App [34] | Germany | GitHub with app algorithms and documentation | Facilitates transparency by allowing public access to app algorithms and documentation | Ethical | Grey literature |
| 35 | O’Callaghan M. et al. (2020) [35] | Ireland | Original research | A national survey of attitudes to digital contact tracing for COVID-19 | Ethical, Privacy and data protection, technical | Indexed literature search |
| 36 | Altmann S. et al. (2020) [36] | France/Germany/Italy/UK/USA | Original research | An international survey of acceptability of app-based contact tracing | Ethical, Privacy and data protection | Indexed literature search |
| 37 | Hassandoust F. et al (2020) [37] | New Zealand/United States of America | Original research | A survey in the USA of privacy concerns with mobile contact tracing apps. | Ethical, Privacy and data protection | Indexed literature search |
| 38 | Ivers L. et al. (2020) [38] | United States of America | Perspective | Discussion of potential utility of digital contact tracing. | Ethical | Indexed literature search |
| 39 | Kirby T. (2020) [39] | N/a | Perspective | Describes effects of COVID-19 on Black, Asian and ethnic minorities | Ethical | Indexed literature search |
| 40 | Armitage R. et al. (2020) [40] | United Kingdom | Perspective | Discusses next consequences of isolation for elderly people. | Ethical | References of included articles |
| 41 | Kumar et al. (2019) [41] | United States of America | Original research | Survey of low-income patients in primary care regarding smartphone ownership and digital literacy. | Ethical | References of included articles |
| 42 | Vokinger K. et al (2020) [42] | Switzerland | Original research | Description of a framework to evaluate digital contact tracing apps from epidemiological and legal perspectives. | Ethical, Privacy and data protection. | Indexed literature search |
| 43 | British Broadcasting Company (2020) [43] | N/a | Press release | Describes the distribution of contact tracing tokens in Singapore. | Ethical | Grey literature review |
| 44 | Anderez D. et al. (2020) [44] | United Kingdom | Original research | Study modelling how a reduction in the  exposure of vulnerable individuals to COVID-19 could minimise the number of deaths. | Ethical | Indexed literature search |
| 45 | Rowe F. (2020) [45] | France | Perspective | Discussion of the long-term implications for privacy due to increased surveillance facilitated by digital contact tracing | Ethical, Privacy and data protection. | Indexed literature search |
| 46 | Couch et al. (2020) [46] | Australia | Perspective | Discussion of the long-term implications for privacy due to increased surveillance facilitated by digital contact tracing | Ethical, Privacy and data protection. | Indexed literature search |
| 47 | Thomas R. et al. (2020)[47] | Australia | Original research | Survey of people’s concerns about downloading national COVID SAFE contact tracing app. | Ethical. Privacy and data protection. | Indexed literature search |
| 48 | von Wyl V. et al (2021) [48] | Switzerland | Original research | Survey of factors influencing acceptability of digital contact tracing app | Ethical, User experience, Privacy and data protection | Indexed literature search |
| 49 | Garrett P. et al. (2021) [49] | Australia | Original research | Survey of acceptability and uptake of smartphone tracking for COVID-19 | Ethical, Privacy and data protection, Technical | Indexed literature search |
| 50 | Zimmermann B. et al (2021) [50] | Germany | Original research | Qualitative interviews and media analysis In German speaking countries to assess early perceptions of digital contact tracing apps | Ethical, Privacy and data protection | Indexed literature search |
| 51 | Nakamoto I. et al. (2020) [51] | Japan | Original research | Description of the deployment of COCOA digital contract tracing app and modelling of its potential impact. | Privacy and data protection, Technical, Clinical and societal | Indexed literature search |
| 52 | Horstmann K. et al. (2021) [52] | Germany | Original research | Survey of reasons for using or not using Corona-Warn app. | User experience | Indexed literature search |
| 53 | Kaspar K. et al. (2020) [53] | Germany | Original research | Survey of factors influencing motivations for social distancing and using a contact tracing app | Ethical | Indexed literature search |
| 54 | Currie D. et al (2020) [54] | Australia | Original research | Modelling study of the potential effectiveness of a digital contact tracing app in containing COVID-19 epidemic. | Ethical | Indexed literature search |
| 55 | Dave R. et al (2020) [55] | India | Perspective | Discussion on how mandatory use of digital contact tracing apps could be ethically justifiable. | Ethical | Indexed literature search |
| 56 | Oldeweme A. et al (2021) [56] | Germany | Original research | Two-point survey (pre and post digital contact tracing app deployment) investigating how uncertainty reduction measures foster the adoption of COVID-19 tracing apps and how their use affects the perception of different risks. | Ethical, Technical | Indexed literature search |
| 57 | Garrett p. et al. (2020) [57] | Taiwan | Original research | Survey of young adults views on smartphone Tracking Technologies | Ethical, Technical | Indexed literature search |
| 58 | Wilmink G. et al. (2020) [58] | United States of America | Original research | Description of a automated digital contact tracing system for long term care facilities and modelling of potential impact on containing COVID-19 outbreaks. | Ethical | Indexed literature search |
| 59 | Goel S. et al. (2018) [59] | N/a | Book chapter | Discusses the factors for the usability of different applications. | User experience | Grey literature |
| 60 | Gelman DL. (2014) [60] | N/a | Book | Design for kids: Digital products for playing and learning. Rosenfeld Media | User experience | Grey literature |
| 61 | Soni N. et al. (2019) [61] | United States of America | Original research | Empirical evaluation of whether evidence-based design recommendations were implemented within apps for children. | User experience | Grey literature |
| 62 | Alonso-Rios D. et al (2009) [62] | Spain | Perspective | Describes the concept of usability and to describe it by means of a detailed taxonomy. | User experience | Grey literature |
| 63 | Kaur E. et al. (2016) [63] | Australia | Perspective | Discusses a user context model and a set of usability attributes for developing mobile applications in healthcare | User experience | Grey literature |
| 64 | Kasali F. et al (2019) [64] | Nigeria | Original research | Describes a usability model to aid the design and assessment of highly usable apps. | User experience | Grey literature |
| 65 | Kascak LR et al. (2014) [65] | United States of America | Literature review | Describes a a set of inclusive design guidelines that reconcile inconsistencies between design guidelines and UD principles. | User experience | Grey literature |
| 66 | Harrington et al. (2017) [66] | United States of America | Perspective | Describes a tool for evaluating mHealth applications for usability and perceived usefulness. | User experience | Grey literature |
| 67 | Ballantyne M. et al (2018) [67] | United States of America | Original research | Analysis of app adherence with accessibility guidelines. | User experience | Grey literature |
| 68 | Staats R. [68] | United States of America | Perspective | Discusses designing user interfaces for color blind users. | User experience | Grey literature |
| 69 | Sanwikarja P. (2020) [69] | Netherlands | Perspective | Discusses design considerations for COVID-19 contact tracing apps | User experience | Grey literature |
| 70 | United Nations Office of the High Commission for Human Rights [70] | N/a | Press release | Statement on the importance of access to and free flow of information during pandemic. | User experience | Grey literature |
| 71 | European Parliament [71] | European Union | Regulation | General Data Protection Regulations | User experience, Privacy and data protection | Grey literature |
| 72 | European Parliament [72] | European Union | Regulation | Directive (EU) 2016/2102 of the European Parliament and of the Council of 26 October 2016 on the accessibility of the websites and mobile applications of public sector bodies | User experience | Grey literature |
| 73 | ETSI [73] | N/a | Standards | Accessibility requirements for ICT products and services | User experience | Grey literature |
| 74 | eHealth Network [74] | European Union | Guidance | Guidance on COVID-19 digital contact tracing app design and deployment | User experience, Privacy and data protection, Technical | Grey literature |
| 75 | Web accessibility initiative [75] | N/a | Guidance | Web Content Accessibility Guidelines | User experience | Grey literature |
| 76 | Xcertia [76] | N/a | Guidance | Guidance on mhealth operability,  privacy, security and content | User experience | Grey literature |
| 77 | Aman [77] | Jordan | App | Digital contact tracing app (GPS) | User experience | Grey literature |
| 78 | Rekanar K. et al (2021) [78] | Ireland | Original research | Analysis of sentiments expressed in user feedback of national contact tracing app. | User experience, Privacy and data protection | Indexed literature search |
| 79 | UK Government Healthtech blog [79] | United Kingdom | Perspective | Update on NHS COVID-19 contact tracing app and a discussion of technical changes to app. | Privacy and data protection | Grey literature |
| 80 | Corona-Warn [80] | Germany | App | Digital contact tracing app (Bluetooth LE) | Ethical, User experience, Privacy and data protection, Technical | Grey literature |
| 81 | NOVID [81] | United States of America | App | Digital contract tracing app  (Bluetooth/Ultrasound/Wi-fi) | User experience, Technical | Grey literature |
| 82 | PathCheck [82] | United States of America | App | Digital contact tracing app  (GPS/Bluetooth LE) | User experience | Grey literature |
| 83 | Stop Covid [83] | Poland | App | Digital contact tracing app  (Bluetooth LE) | User experience | Grey literature |
| 84 | Storni C. et al. (2020) [84] | Ireland | Original research | Describes the  development of a usability pillar for an evaluation taxonomy of COVID-19 digital contact tracing apps | User experience | Grey literature |
| 85 | COVID Tracker [85] | Ireland | Report | Data protection impact assessment | Privacy and data protection | Grey literature |
| 86 | Tosi J. et al. (2017) [86] | Italy | Systematic review | Reviews the state of the art on Bluetooth LE characteristics and performance. | Technical | References of included articles |
| 87 | Gibney S. et al. (2020) [87] | Ireland | Report | Describes the profile of smartphone ownership and use in Ireland. | User experience, Technical | Grey literature |
| 88 | European Data Protection Board (2020) [88] | European Union | Guidance | Guidelines on the use of location data and contact tracing tools in the context of the COVID-19 outbreak. | Privacy and data protection | Grey literature |
| 89 | Cavoukian A. et al (2009) [89] | Canada | Guidance | Describes foundational principles of privacy preservation | Privacy and data protection | References of included articles |
| 90 | Keshet Y. (2020) [90] | Israel | Original research | Qualitative analysis of comments on articles about automated contact tracing on news sites | Privacy and data protection | Indexed literature search |
| 91 | Lauristin M. (2017) [91] | European Union | Report | Describes regulation regarding the protection of personal data in electronic communications | Privacy and data protection | References of included articles |
| 92 | Data Protection Commission [92] | Ireland | Guidance | Provides guidance on the legal bases for processing personal data | Privacy and data protection | Grey literature |
| 93 | Data Protection Commission [93] | Ireland | Report | Describes the principles of data protection | Privacy and data protection | Grey literature |
| 94 | Gibney S. [94] | Ireland | Original research | Describes user perspectives and experiences with COVID Tracker app. | Privacy and data protection | Grey literature |
| 95 | Loh P. [95] | United States of America | Original research | Evaluating the accuracy of NOVD app | Privacy and data protection, Technical | Grey literature |
| 96 | Leith DJ. et al. (2020) [96] | Ireland | Original research | Evaluating the Google/Apple COVID exposure notification API | Privacy and data protection | Indexed literature search |
| 97 | Leith DJ. et al. (2020) [97] | Ireland | Original research | Measurement-based evaluation of google/apple exposure notification api for proximity detection in a commuter bus | Privacy and data protection, Technical | Indexed literature search |
| 98 | Park S. et al. (2020) [98] | South Korea | Perspective | Description of contact tracing in South Korea including strengths and weaknesses | Privacy and data protection | Indexed literature search |
| 99 | Bengio et al. (2021) [99] | Canada/United States of America | Perspective | Discusses privacy limitations of any decentralized automatic contact tracing system. | Privacy and data protection, Technical | Indexed literature search |
| 100 | Patil S. et al. (2016) [100] | Europe | Original research |  | Privacy and data protection | References of included articles |
| 101 | Park O. et al. (2020) [101] | South Korea | Review | Review of South Korean COVID-19 contact tracing experience | Privacy and data protection, Technical | Indexed literature search |
| 102 | New York Times (2018) [102] | United States of America | Press release | Describes privacy and data protection risks associated with mobile app use. | Privacy and data protection | Grey literature |
| 103 | The Guardian [103] | United Kingdom | Press release | Discussion of privacy concerns surrounding automated contact tracing for COVID-19 in South Korea | Privacy and data protection | Grey literature |
| 104 | Renew Europe [104] | Europe | Webinar | Webinar discussing the design and deployment of digital contact tracing apps for COVID-19 outbreak control | Privacy, data protection, Technical | Grey literature |
| 105 | Amit et al. (2020) [105] | Israel | Perspective | Discussion of the use of mass-surveillance technology in Israel to contain COVID-19 epidemic. | Privacy and data protection, Technical | Grey literature |
| 106 | Barth S. et al. (2017) [106] | Netherlands | Systematic review | Discusses evidence regarding the privacy paradox | Privacy and data protection | References of included articles |
| 107 | Walrave M. et al. (2020)  [107] | Belgium | Original research | Survey of 1500 people in Belgium of factors influencing app use intention. | Privacy and data protection | Indexed literature search |
| 108 | Guillion M. et al. (2020) [108] | France | Original research | Survey of support for quarantine and digital contact tracing in France. | Ethical | Indexed literature search |
| 109 | Bachtiger P. et al. (2020) [109] | United Kingdom | Original research | Survey of willingness to participate in app based contact tracing | Privacy and data protection | Indexed literature search |
| 110 | Zhang B. et al. (2020) [110] | United States of America | Original research | Survey of peoples perceptions of privacy and surveillance in the COVID-19 pandemic | Privacy and data protection | Indexed literature search |
| 111 | Scherr T. et al. (2021) [111] | United States of America | Original research | Description of barcode-based digital contact tracing app use and survey of users impressions of usability. | Technical | Indexed literature search |
| 112 | Jonker M. et al (2020) [112] | Netherlands | Original research | Description of factors influencing willingness to use digital contact tracing app. | Privacy and data protection | Indexed literature search |
| 113 | Bradshaw W. et al. (2021)  [113] | United States of America | Original research | Modelling study describing the potential for unidirectional and bidirectional digital contact tracing to contain COVID-19 epidemics. | Technical | Indexed literature search |
| 114 | Zhang M. et al. (2020) [114] | Singapore | Original research | Evaluation of the readability of COVID-19 contact tracing app privacy agreements | Privacy and data protection | Indexed literature search |
| 115 | Lewandowsky S. et al. (2021) [115] | United Kingdom | Original research | Survey of people’s acceptance of different digital contact tracing apps and different strategies such as sunset clauses. | Privacy and data protection | Indexed literature search |
| 116 | Nguyen CT. et al (2020) [116] | Australia/Luxembourg | Original research | An evaluation of how emerging technologies can enable, encourage, and enforce social distancing practice. | Technical, Evaluation | Indexed literature search |
| 117 | Simko L. et al. (2020) [117] | United States of America | Original research | A survey of public opinion on the balance between effective technology-based contact tracing and the privacy of individuals | Technical | Preprint server |
| 118 | Huang Z. et al. (2020) [118] | Singapore | Original research | Comparison of TraceTogether—with that of a wearable tag-based real-time locating system | Technical | Indexed literature search |
| 119 | Meyer S. et al. (2020) [119] | Germany | Original research | An evaluation of the Google exposure notification API | Technical | Grey literature |
| 120 | Liu S. et al. (2011) [120] | United States of America | Original research | Experimental  Studies assessing the efficacy of Bluetooth in measuring proximity | Technical | Grey literature |
| 121 | Bay J. et al. (2020)  [121] | Singapore | Report | Description of BlueTrace privacy preserving protocol. | Technical | Grey literature |
| 122 | Stanley J. et al. (2020)  [122] | United States of America | Report | Discusses limitations of location tracing for digital contact tracing in COVID-19. | Technical | Grey literature |
| 123 | Salathé M. et al. (2020) [123] | Switzerland | Original research | Evaluation of digital contact tracing app effectiveness | Technical, Evaluation | Indexed literature search |
| 124 | Rodriguez P. et al. (2021) [124] | Spain | Original research | A population-based controlled experiment assessing the epidemiological impact of digital contact tracing | Technical, Evaluation | Indexed literature search |
| 125 | Association of State and Territorial Health Officials [125] | United States of America | Report | Describes considerations when using digital contact technologies to contain COVID-19 | Technical considerations | Grey literature |
| 126 | Loh P. (2020) [126] | United States of America | Perspective | Discussion on approaches to digital contact tracing | Technical | Preprint server |
| 127 | Nearform [127] | Ireland | Website | Description of Bluetooth LE digital contact tracing | Technical | Grey literature |
| 128 | Troncoso et al. (2020) [128] | N/a | White paper | Decentralized Privacy-Preserving Proximity Tracing | Technical | Grey literature |
| 129 | Pan-European Privacy-Preserving Proximity Tracing [129] | N/a | Website | Pan-European Privacy-Preserving Proximity Tracing | Technical | Grey literature |
| 130 | Centre for Disease Control [130] | United States of America | Guidance | Guidance on contact tracing for COVID-19 | Technical | Grey literature |
| 131 | European Centre for Disease Control [131] | Europe | Guidance | Guidance on contact tracing for COVID-19 | Technical, Evaluation | Grey literature |
| 132 | Qureshi Z. et al. (2020) [132] | United Kingdom | Review | Evidence synthesis regarding 2m social distancing guidelines. | Technical | Grey literature |
| 133 | Corona-Warn team [133] | Germany | Report | This document contains an epidemiological description of the transmission risk level used in the German  Corona-Warn-App. | Technical | Grey literature |
| 134 | Jehi L. [134] | United States of America | Original research | Individualizing Risk Prediction for Positive Coronavirus Disease 2019 Testing | Clinical and societal | References of included studies |
| 135 | Munzert S. et al. (2021) [135] | Germany | Original research | Combined study of online survey with mobile tracking data to evaluate interventions to increase digital contact tracing app uptake. | Clinical and societal | Indexed literature search |
| 136 | Ktretschmar M. et al. ([136]) | Europe | Original research | Modelling study of the potential impact different contact tracing strategies could have on a COVID-19 epidemic. | Clinical and societal, Evaluation | Indexed literature search |
| 137 | Yasaka T. et al. (2020) [137] | Unites States of America | Original research | Description and modelling study of a digital contact tracing app that uses QR code scanning to create “checkpoints”. | Clinical and societal | Indexed literature search |
| 138 | Young ES. Et al. (2021) [138] | Singapore | Original research | Nationwide survey to determine behavioural and demographic characteristics associated with TraceTogether uptake. | Clinical and societal | Indexed literature search |
| 139 | Wall Street Journal [139] | China | Press release | Discusses COVID-19 outbreak containment measures in China | Evaluation | Grey literature |
| 140 | Nature [140] | N/a | Perspective | Discussion of recent studies evaluating digital contact tracing app performance | Evaluation | Indexed literature search |
| 141 | Menges D. et al. [141] | Switzerland | Original research | Description of cascade of use and contact notification of digital contact tracing app. | Evaluation | References of included studies |
| 142 | Ballouz T. et al. (2020) [142] | Switzerland | Original research | Description of cascade of use and contact notification of digital contact tracing app. | Evaluation | References of included studies |
| 143 | Masel J. et al. (2021) [143] | United States of America | Original research | Survey of COVID-19 cases of digital contact tracing app and contact alert use to evaluate app performance. | Evaluation | References of included studies |
| 144 | Wymant et al. (2021) [144] | United Kingdom | Original research | Description of UK NHSX digital contact tracing app performance | Evaluation | References of included studies |
| 145 | Almagor J. et al. (2020) [145] | United Kingdom | Original research | Modelling study of the potential impact of a digital contact tracing app in containing a COVID-19 epidemic. | Evaluation | Indexed literature search |

References

1. Scottish Human Rights Commission. COVID – 19: Human Rights implications of digital contact tracing technology. Scottish Human Rights Commission. 2020;

2. Ada Lovelace institute. Exit through the App Store? Rapid Evidence Review, April 2020. [cited 2021 Feb 4]; Available from: https://www.adalovelaceinstitute.org/wp-content/uploads/2020/04/Ada-Lovelace-Institute-Exit-through-the-App-Store-Explainer-for-Government-April-2020.pdf

3. Ferretti L, Wymant C, Kendall M, Zhao L, Nurtay A, Abeler-Dӧrner L, et al. Quantifying SARS-CoV-2 transmission suggests epidemic control with digital contact tracing. Science. American Association for the Advancement of Science; 2020;368(6491).

4. COVID-19 digital apps need due diligence [Internet]. Nature. 2020 [cited 2021 Feb 4]. Available from: https://media.nature.com/original/magazine-assets/d41586-020-01264-1/d41586-020-01264-1.pdf

5. Upshur RE. Principles for the justification of public health intervention. Canadian journal of public health. Springer; 2002;93(2):101–3.

6. Childress JF, Faden RR, Gaare RD, Gostin LO, Kahn J, Bonnie RJ, et al. Public health ethics: mapping the terrain. Arguing about bioethics London \& New York: Routledge. 2012;361–73.

7. Kass NE. An ethics framework for public health. American journal of public health. American Public Health Association; 2001;91(11):1776–82.

8. Parker MJ, Fraser C, Abeler-Dӧrner L, Bonsall D. Ethics of instantaneous contact tracing using mobile phone apps in the control of the COVID-19 pandemic. Journal of Medical Ethics. Institute of Medical Ethics; 2020;

9. World Health Organization. Ethical considerations to guide the use of digital proximity tracking technologies for COVID-19 contact tracing: interim guidance. 28 May 2020. [cited 2021 Mar 17]; Available from: https://ec.europa.eu/commission/presscorner/detail/en/IP_20_626

10. World Health Organisation. Contact tracing in the context of COVID-19: interim guidance, 10 May 2020. 2020;

11. Ranisch R, Nijsingh N, Ballantyne A, van Bergen A, Buyx A, Friedrich O, et al. Digital contact tracing and exposure notification: ethical guidance for trustworthy pandemic management. Ethics and information technology. Springer; 2020;1–10.

12. Lockey S, Edwards MR, Hornsey MJ, Gillespie N, Akhlaghpour S, Colville S. Profiling adopters (and non-adopters) of a contact tracing mobile application: Insights from Australia. International journal of medical informatics. Elsevier; 2021;104414.

13. Gasser U, Ienca M, Scheibner J, Sleigh J, Vayena E. Digital tools against COVID-19: taxonomy, ethical challenges, and navigation aid. The Lancet Digital Health. Elsevier; 2020;

14. Lo B, Sim I. Ethical Framework for Assessing Manual and Digital Contact Tracing for COVID-19. Annals of Internal Medicine. American College of Physicians; 2020;

15. Morley J, Cowls J, Taddeo M, Floridi L. Ethical guidelines for COVID-19 tracing apps. Nature Publishing Group; 2020;

16. Coronavirus: Commission adopts Recommendation to support exit strategies through mobile data and apps. Press release. European Commission, April 8th 2020.

17. Nuffield Council on Bioethics. Ethical considerations in responding to the COVID-19 pandemic. March 17th 2020. [Internet]. [cited 2021 Mar 17]. Available from: https://www.nuffieldbioethics.org/publications/ethical-considerations-in-responding-to-the-covid-19-pandemic

18. Sharon T. Blind-sided by privacy? Digital contact tracing, the Apple/Google API and big tech’s newfound role as global health policy makers. Ethics and Information Technology. Springer; 2020;1–13.

19. Storeng KT, de Bengy Puyvallée A. The Smartphone Pandemic: How Big Tech and public health authorities partner in the digital response to Covid-19. Global Public Health. Taylor \& Francis; 2021;1–17.

20. Williams SN, Armitage CJ, Tampe T, Dienes K. Public attitudes towards COVID-19 contact tracing apps: A UK-based focus group study. Health Expectations. Wiley Online Library; 2020;

21. Braithwaite I, Callender T, Bullock M, Aldridge RW. Automated and partly automated contact tracing: a systematic review to inform the control of COVID-19. The Lancet Digital Health. Elsevier; 2020;

22. Anglemyer A, Moore TH, Parker L, Chambers T, Grady A, Chiu K, et al. Digital contact tracing technologies in epidemics: a rapid review. Cochrane Database of Systematic Reviews. John Wiley \& Sons, Ltd; 2020;(8).

23. Shamil MS, Farheen F, Ibtehaz N, Khan IM, Rahman MS. An Agent-Based Modeling of COVID-19: Validation, Analysis, and Recommendations. Cognitive Computation. Springer; 2021;1–12.

24. Von Wyl V, Bonhoeffer S, Bugnion E, Puhan MA, Salathé M, Stadler T, et al. A research agenda for digital proximity tracing apps. Swiss medical weekly. 2020;150:w20324.

25. Roderick P, Macfarlane A, Pollock AM. Getting back on track: control of covid-19 outbreaks in the community. bmj. British Medical Journal Publishing Group; 2020;369.

26. Contact Tracing Is Failing in Many States. Here’s Why. July 21st 2020. 2020 [cited 2021 Jan 25]; Available from: https://www.nytimes.com/2020/07/31/health/covid-contact-tracing-tests.html

27. Baumgärtner L, Dmitrienko A, Freisleben B, Gruler A, Hӧchst J, Kühlberg J, et al. Mind the GAP: Security \& Privacy Risks of Contact Tracing Apps. arXiv preprint arXiv:200605914. 2020;

28. Iacobucci G. Government’s plan to digitise NHS risks wasting billions, MPs warn. British Medical Journal Publishing Group; 2020 [cited 2021 Feb 4]; Available from: https://www.bmj.com/content/371/bmj.m4317

29. Von Wyl V. Challenges for Nontechnical Implementation of Digital Proximity Tracing During the COVID-19 Pandemic: Media Analysis of the SwissCovid App. JMIR mHealth and uHealth. JMIR Publications Inc., Toronto, Canada; 2021;9(2):e25345.

30. Berman G, Carter K, Herranz MG, Sekara V, others. Digital contact tracing and surveillance during COVID-19. General and child-specific ethical issues. United Nations International Children’s Emergency Fund. 2020.

31. Leith DJ, Farrell S. Measurement-based evaluation of Google/Apple Exposure Notification API for proximity detection in a light-rail tram. Plos one. Public Library of Science San Francisco, CA USA; 2020;15(9):e0239943.

32. European Centre for Disease Control. Mobile applications in support of contact tracing for COVID-19, 2020. [cited 2021 Feb 4]; Available from: https://www.ecdc.europa.eu/sites/default/files/documents/covid-19-mobile-applications-contact-tracing.pdf

33. GitHub COVID Tracker Health Service Executive . [cited 2021 Jan 25]; Available from: https://github.com/HSEIreland/

34. GitHub Corona-Warn-App. [cited 2021 Jan 25]; Available from: https://github.com/corona-warn-app

35. O’Callaghan ME, Buckley J, Fitzgerald B, Johnson K, Laffey J, McNicholas B, et al. A national survey of attitudes to COVID-19 digital contact tracing in the Republic of Ireland. Irish Journal of Medical Science (1971-). Springer; 2020;1–25.

36. Altmann S, Milsom L, Zillessen H, Blasone R, Gerdon F, Bach R, et al. Acceptability of app-based contact tracing for COVID-19: Cross-country survey evidence. Available at SSRN 3590505. 2020;

37. Hassandoust F, Akhlaghpour S, Johnston AC. Individuals’ privacy concerns and adoption of contact tracing mobile applications in a pandemic: A situational privacy calculus perspective. Journal of the American Medical Informatics Association. 2020;

38. Ivers LC, Weitzner DJ. Can digital contact tracing make up for lost time? The Lancet Public Health. Elsevier; 2020;5(8):e417–e418.

39. Kirby T. Evidence mounts on the disproportionate effect of COVID-19 on ethnic minorities. The Lancet Respiratory Medicine. Elsevier; 2020;8(6):547–8.

40. Armitage R, Nellums LB. COVID-19 and the consequences of isolating the elderly. The Lancet Public Health. Elsevier; 2020;5(5):e256.

41. Kumar D, Hemmige V, Kallen MA, Giordano TP, Arya M. Mobile phones may not bridge the digital divide: a look at mobile phone literacy in an underserved patient population. Cureus. Cureus Inc.; 2019;11(2).

42. Vokinger KN, Nittas V, Witt CM, Fabrikant SI, von Wyl V. Digital health and the COVID-19 epidemic: an assessment framework for apps from an epidemiological and legal perspective. Swiss Medical Weekly. EMH Swiss Medical Publishers; 2020;150:w20282.

43. Singapore distributes Covid contact-tracing tokens [Internet]. [cited 2020 Nov 13]. Available from: https://www.bbc.com/news/business-54143015#:~:text=Singapore%20has%20started%20distributing%20Bluetooth,was%20rolled%20out%20in%20March.

44. Anderez DO, Kanjo E, Pogrebna G, Kaiwartya O, Johnson SD, Hunt JA. A COVID-19-based modified epidemiological model and technological approaches to help vulnerable individuals emerge from the lockdown in the UK. Sensors. Multidisciplinary Digital Publishing Institute; 2020;20(17):4967.

45. Rowe F. Contact tracing apps and values dilemmas: A privacy paradox in a neo-liberal world. International Journal of Information Management. Elsevier; 2020;55:102178.

46. Couch DL, Robinson P, Komesaroff PA. COVID-19—extending surveillance and the panopticon. Journal of bioethical inquiry. Springer; 2020;17(4):809–14.

47. Thomas R, Michaleff ZA, Greenwood H, Abukmail E, Glasziou P. Concerns and Misconceptions About the Australian Government’s COVIDSafe App: Cross-Sectional Survey Study. JMIR public health and surveillance. JMIR Publications Inc., Toronto, Canada; 2020;6(4):e23081.

48. Von Wyl V, Hӧglinger M, Sieber C, Kaufmann M, Moser A, Serra-Burriel M, et al. Drivers of acceptance of COVID-19 proximity tracing apps in Switzerland: panel survey analysis. JMIR public health and surveillance. JMIR Publications Inc., Toronto, Canada; 2021;7(1):e25701.

49. Garrett PM, White JP, Lewandowsky S, Kashima Y, Perfors A, Little DR, et al. The acceptability and uptake of smartphone tracking for COVID-19 in Australia. PloS one. Public Library of Science San Francisco, CA USA; 2021;16(1):e0244827.

50. Zimmermann BM, Fiske A, Prainsack B, Hangel N, McLennan S, Buyx A. Early Perceptions of COVID-19 Contact Tracing Apps in German-Speaking Countries: Comparative Mixed Methods Study. Journal of medical Internet research. JMIR Publications Inc., Toronto, Canada; 2021;23(2):e25525.

51. Nakamoto I, Jiang M, Zhang J, Zhuang W, Guo Y, Jin M-H, et al. Evaluation of the Design and Implementation of a Peer-To-Peer COVID-19 Contact Tracing Mobile App (COCOA) in Japan. JMIR Mhealth Uhealth. 2020;e22098–e22098.

52. Horstmann KT, Buecker S, Krasko J, Kritzler S, Terwiel S. Who does or does not use the “Corona-Warn-App”and why? European Journal of Public Health. Oxford University Press; 2021;31(1):49–51.

53. Kaspar K. Motivations for social distancing and app use as complementary measures to combat the COVID-19 pandemic: quantitative survey study. Journal of medical Internet research. JMIR Publications Inc., Toronto, Canada; 2020;22(8):e21613.

54. Currie DJ, Peng CQ, Lyle DM, Jameson BA, Frommer MS. Stemming the flow: how much can the Australian smartphone app help to control COVID-19. Public Health Res Pract. 2020;30(2):e3022009.

55. Dave R, Gupta R. Mandating the Use of Proximity Tracking Apps During Coronavirus Disease 2019: Ethical Justifications. Frontiers in Medicine. Frontiers Media SA; 2020;7.

56. Oldeweme A, Märtins J, Westmattelmann D, Schewe G. The Role of Transparency, Trust, and Social Influence on Uncertainty Reduction in Times of Pandemics: Empirical Study on the Adoption of COVID-19 Tracing Apps. Journal of medical Internet research. JMIR Publications Inc., Toronto, Canada; 2021;23(2):e25893.

57. Garrett PM, Wang Y, White JP, Hsieh S, Strong C, Lee Y-C, et al. Young adults view smartphone tracking technologies for COVID-19 as acceptable: the case of Taiwan. International journal of environmental research and public health. Multidisciplinary Digital Publishing Institute; 2021;18(3):1332.

58. Wilmink G, Summer I, Marsyla D, Sukhu S, Grote J, Zobel G, et al. Real-time digital contact tracing: development of a system to control COVID-19 outbreaks in nursing homes and long-term care facilities. JMIR Public Health and Surveillance. JMIR Publications Inc., Toronto, Canada; 2020;6(3):e20828.

59. Goel S, Nagpal R, Mehrotra D. Mobile applications usability parameters: Taking an insight view. Information and Communication Technology for Sustainable Development. Springer; 2018. p. 35–43.

60. Gelman DL. Design for kids: Digital products for playing and learning. Rosenfeld Media; 2014.

61. Soni N, Aloba A, Morga KS, Wisniewski PJ, Anthony L. A framework of touchscreen interaction design recommendations for children (tidrc) characterizing the gap between research evidence and design practice. Proceedings of the 18th ACM International Conference on Interaction Design and Children. 2019. p. 419–31.

62. Alonso-Rios D, Vázquez-Garcia A, Mosqueira-Rey E, Moret-Bonillo V. Usability: a critical analysis and a taxonomy. International journal of human-computer interaction. Taylor \& Francis; 2009;26(1):53–74.

63. Kaur E, Haghighi PD. A context-aware usability model for mobile health applications. Proceedings of the 14th International Conference on Advances in Mobile Computing and Multi Media. 2016. p. 181–9.

64. Kasali F, Taiwo O, Akinyemi I, Alaba O, Awodele O, Kuyoro S. An Enhanced Usability Model for Mobile Health Application. International Journal of Computer Science and Information Security (IJCSIS). 2019;17(2).

65. Kascak LR, Rébola CB, Sanford JA. Integrating Universal Design (UD) principles and mobile design guidelines to improve design of mobile health applications for older adults. 2014 IEEE International Conference on Healthcare Informatics. 2014. p. 343–8.

66. Harrington CN, Ruzic L, Sanford JA. Universally accessible mHealth apps for older adults: Towards increasing adoption and sustained engagement. International Conference on Universal Access in Human-Computer Interaction. 2017. p. 3–12.

67. Ballantyne M, Jha A, Jacobsen A, Hawker JS, El-Glaly YN. Study of accessibility guidelines of mobile applications. Proceedings of the 17th international conference on mobile and ubiquitous multimedia. 2018. p. 305–15.

68. Staats R. Designing UI with Color Blind Users in Mind. 2020 [cited 2021 Jan 23]; Available from: https://www.secretstache.com/blog/designing-for-color-blind-users/

69. Sanwikarja P. Contact tracing: How do you design an app millions of people will trust? 2020 [cited 2021 Jan 23]; Available from: https://uxdesign.cc/how-do-you-design-an-app-millions-of-people-will-trust-8a63f5a5660a

70. COVID-19: Governments must promote and protect access to and free flow of information during pandemic – International experts. 2020 [cited 2021 Jan 23]; Available from: https://www.ohchr.org/en/NewsEvents/Pages/DisplayNews.aspx?NewsID=25729&LangID=E.

71. General Data Protection Regulation EU 2016/679 of the European Parliament and of the Council of 27 April 2016. Official Journal of the European Union Available at: http://ec europa eu/justice/data-protection/reform/files/regulation\_oj\_en pdf (accessed 20 September 2017). 2016;

72. Directive (EU) 2016/2102 of the European Parliament and of the Council of 26 October 2016 on the accessibility of the websites and mobile applications of public sector bodies. Eur-Lex [Internet]. [cited 2021 Mar 18]. Available from: https://eur-lex.europa.eu/legal-content/EN/TXT/?uri=CELEX%3A32016L2102

73. ETSI. Accessibility requirements for ICT products and services,” vol. 2, pp. 1–152, 2018. 2018 [cited 2021 Jan 23]; Available from: https://www.etsi.org/deliver/etsi_en/301500_301599/301549/02.01.02_60/en_301549v020102p.pdf

74. Mobile Applications to support contact tracing in the EU’s fight against COVID-19. Common EU Toolbox for Member States,European eHealth Network, 2020;1-56.

75. Web Content Accessibility Guidelines (WCAG) Overview [Internet]. 2020 [cited 2021 Jan 23]. Available from: https://www.w3.org/WAI/standards-guidelines/wcag/

76. Xcertia mHealth App Guidelines [Internet]. 2019 [cited 2021 Jan 23]. Available from: https://www.himss.org/sites/hde/files/media/file/2020/04/17/xcertia-guidelines-2019-final.pdf

77. Aman app [Internet]. 2020 [cited 2021 Jan 23]. Available from: https://amanapp.jo/en

78. Rekanar K, O’Keeffe IR, Buckley S, Abbas M, Beecham S, Chochlov M, et al. Sentiment analysis of user feedback on the HSE’s Covid-19 contact tracing app. Irish Journal of Medical Science (1971-). Springer; 2021;1–10.

79. How the NHS COVID-19 app is making the most of cutting-edge global technology [Internet]. [cited 2020 Nov 28]. Available from: https://healthtech.blog.gov.uk/2020/10/29/how-the-nhs-covid-19-app-is-making-the-most-of-cutting-edge-global-technology/

80. Corona-Warn-App [Internet]. 2020 [cited 2021 Jan 23]. Available from: https://www.coronawarn.app/en/

81. NOVID app [Internet]. 2020 [cited 2020 Nov 26]. Available from: https://www.novid.org/#howitworks

82. PathCheck app. 2020 [cited 2021 Jan 23]; Available from: https://www.pathcheck.org/

83. Stop COVID app. 2020 [cited 2021 Jan 23]; Available from: https://www.gov.pl/web/protegosafe

84. Storni C., Tsvyatkova D., Richardson I., Buckley J., Abbas M. et al. Toward a Compare and Contrast Framework for COVID-19 Contact Tracing Mobile Applications: a Look at Usability HealthINF Conference . 2021.

85. Data Protection Impact Assessment- COVID Tracker App. Department of Health, Government of Ireland, 2020.

86. Tosi J, Taffoni F, Santacatterina M, Sannino R, Formica D. Performance evaluation of bluetooth low energy: A systematic review. Sensors. Multidisciplinary Digital Publishing Institute; 2017;17(12):2898.

87. Gibney S, McCarthy T. Profile of Smartphone Ownership and Use [Internet]. 2020. Available from: https://assets.gov.ie/81401/e6c10ac5-e6b4-438e-b31d-30cd2b241f7c.pdf

88. Guidelines on the use of location data and contact tracing tools in the context of the COVID-19 outbreak. European Data Protection Board, 2020 .

89. Cavoukian A., Privacy by design: The 7 foundational principles. Information and privacy commissioner of Ontario, Canada [Internet]. [cited 2021 Mar 18]. Available from: https://www.ipc.on.ca/wp-content/uploads/resources/7foundationalprinciples.pdf

90. Keshet Y. Fear of panoptic surveillance: using digital technology to control the COVID-19 epidemic. Israel journal of health policy research. Springer; 2020;9(1):1–8.

91. Lauristin M. Draft Report on the proposal for a regulation of the European Parliament and of the Council concerning the respect for private life and the protection of personal data in electronic communications and repealing Directive 2002/58/EC (Regulation on Privacy and Electronic Communications). European Parliament, Sep. 2017;

92. Data Protection Commission. Guidance Note: Legal Bases for Processing Personal Data. Data Protection Commission, December 2019. [Internet]. [cited 2021 Mar 18]. Available from: https://www.dataprotection.ie/sites/default/files/uploads/2020-04/Guidance%20on%20Legal%20Bases.pdf

93. Principles of Data Protection [Internet]. [cited 2020 Sep 26]. Available from: https://www.dataprotection.ie/en/individuals/principles-data-protection

94. Gibney S, Bruton L, Doherty P. COVID Contact Tracing App: User Perspectives and Experience Research. Research Services and Policy Unit, Research and Development and Health Analytics Division, Department of Health. 2020;

95. Loh P. Accuracy of bluetooth-ultrasound contact tracing: experimental results from NOVID iOS Version 2.1 Using 5-Year-Old Phones. 2020.

96. Leith DJ, Farrell S. Gaen due diligence: Verifying the Google/Apple COVID exposure notification API. CoronaDef21, Proceedings of NDSS ‘21. 2021;

97. Leith DJ, Farrell S. Measurement-based evaluation of google/apple exposure notification api for proximity detection in a commuter bus. arXiv preprint arXiv:200608543. 2020;

98. Park S, Choi GJ, Ko H. Information technology-based tracing strategy in response to COVID-19 in South Korea—privacy controversies. Jama. American Medical Association; 2020;323(21):2129–30.

99. Bengio Y, Ippolito D, Janda R, Jarvie M, Prud’homme B, Rousseau J-F, et al. Inherent privacy limitations of decentralized contact tracing apps. Journal of the American Medical Informatics Association. Oxford University Press; 2021;28(1):193–5.

100. Patil S, Lu H, Saunders CL, Potoglou D, Robinson N. Public preferences for electronic health data storage, access, and sharing—Evidence from a pan-European survey. Journal of the American Medical Informatics Association. Oxford University Press; 2016;23(6):1096–106.

101. Park O, Park YJ, Park SY, Kim YM, Kim J, Lee J, et al. Contact transmission of Covid-19 in South Korea: Novel investigation techniques for tracing contacts. Osong Public Health and Research Perspectives. 2020;(1).

102. Your Apps Know Where You Were Last Night, and They’re Not Keeping It Secret [Internet]. [cited 2021 Jan 13]. Available from: https://www.nytimes.com/interactive/2018/12/10/business/location-data-privacy-apps.html

103. “More scary than coronavirus”: South Korea’s health alerts expose private lives [Internet]. [cited 2020 Nov 12]. Available from: https://www.theguardian.com/world/2020/mar/06/more-scary-than-coronavirus-south-koreas-health-alerts-expose-private-lives

104. RENEW EUROPE Webinar on COVID-19 contact tracing applications [Internet]. [cited 2020 Sep 30]. Available from: https://re.livecasts.eu/webinar-on-contact-tracing-applications/

105. Amit M, Kimhi H, Bader T, Chen J, Glassberg E, Benov A. Mass-surveillance technologies to fight coronavirus spread: the case of Israel. Nature Medicine. Nature Publishing Group; 2020;1–3.

106. Barth S, De Jong MD. The privacy paradox-Investigating discrepancies between expressed privacy concerns and actual online behavior-A systematic literature review. Telematics and informatics. Elsevier; 2017;34(7):1038–58.

107. Walrave M, Waeterloos C, Ponnet K. Adoption of a contact tracing app for containing COVID-19: a health belief model approach. JMIR public health and surveillance. JMIR Publications Inc., Toronto, Canada; 2020;6(3):e20572.

108. Guillon M, Kergall P. Attitudes and opinions on quarantine and support for a contact-tracing application in France during the COVID-19 outbreak. Public health. Elsevier; 2020;188:21–31.

109. Bachtiger P, Adamson A, Quint JK, Peters NS. Belief of having had unconfirmed Covid-19 infection reduces willingness to participate in app-based contact tracing. NPJ digital medicine. Nature Publishing Group; 2020;3(1):1–7.

110. Zhang B, Kreps S, McMurry N, McCain RM. Americans’ perceptions of privacy and surveillance in the COVID-19 pandemic. Plos one. Public Library of Science San Francisco, CA USA; 2020;15(12):e0242652.

111. Scherr TF, DeSousa J, Moore C, Hardcastle A, Wright DW. App Usage and Usability Impressions of a Barcode-Based Digital Contact Tracing Platform for COVID-19: Survey Study. JMIR public health and surveillance. 2021;

112. Jonker M, de Bekker-Grob E, Veldwijk J, Goossens L, Bour S, Rutten-Van Mӧlken M. COVID-19 Contact Tracing Apps: Predicted Uptake in the Netherlands Based on a Discrete Choice Experiment. JMIR mHealth and uHealth. JMIR Publications Inc., Toronto, Canada; 2020;8(10):e20741.

113. Bradshaw WJ, Alley EC, Huggins JH, Lloyd AL, Esvelt KM. Bidirectional contact tracing dramatically improves COVID-19 control. medRxiv. Cold Spring Harbor Laboratory Press; 2020;

114. Zhang M, Chow A, Smith H. COVID-19 Contact-Tracing Apps: Analysis of the Readability of Privacy Policies. Journal of Medical Internet Research. JMIR Publications Inc., Toronto, Canada; 2020;22(12):e21572.

115. Lewandowsky S, Dennis S, Perfors A, Kashima Y, White JP, Garrett P, et al. Public acceptance of privacy-encroaching policies to address the COVID-19 pandemic in the United Kingdom. PloS one. Public Library of Science San Francisco, CA USA; 2021;16(1):e0245740.

116. Nguyen CT, Saputra YM, Van Huynh N, Nguyen N-T, Khoa TV, Tuan BM, et al. Enabling and emerging technologies for social distancing: A comprehensive survey. arXiv preprint arXiv:200502816. 2020;

117. Simko L, Chang JL, Jiang M, Calo R, Roesner F, Kohno T. COVID-19 Contact Tracing and Privacy: A Longitudinal Study of Public Opinion. arXiv preprint arXiv:201201553. 2020;

118. Huang Z, Guo H, Lee Y-M, Ho EC, Ang H, Chow A. Performance of Digital Contact Tracing Tools for COVID-19 Response in Singapore: Cross-Sectional Study. JMIR mHealth and uHealth. JMIR Publications Inc., Toronto, Canada; 2020;8(10):e23148.

119. Meyer S. Google Exposure Notification API Testing Fraunhofer IIS. 2020 [cited 2021 Jan 28]; Available from: https://github.com/corona-warn-app/cwa-documentation/blob/master/2020_06_24_Corona_API_measurements.pdf

120. Liu S, Striegel A. Accurate Extraction of Face-to-Face Proximity Using Smartphones and Bluetooth. 2011 Proceedings of 20th International Conference on Computer Communications and Networks (ICCCN). 2011;1–5.

121. Bay J, Kek J, Tan A, Hau CS, Yongquan L, Tan J, et al. BlueTrace: A privacy-preserving protocol for community-driven contact tracing across borders. Government Technology Agency-Singapore, Tech Rep. 2020;

122. Stanley J, Granick JS. The limits of location tracking in an epidemic. American Civil Liberties Union 2020. [cited 2021 Feb 4]; Available from: https://www.aclu.org/sites/default/files/field_document/limits_of_location_tracking_in_an_epidemic.pdf

123. Salathé M, Althaus C, Anderegg N, Antonioli D, Ballouz T, Bugnon E, et al. Early evidence of effectiveness of digital contact tracing for SARS-CoV-2 in Switzerland. Swiss Medical Weekly. EMH Media; 2020;150(5152).

124. Rodriguez P, Graña S, Alvarez-León EE, Battaglini M, Darias FJ, Hernán MA, et al. A population-based controlled experiment assessing the epidemiological impact of digital contact tracing. Nature Communications. Nature Publishing Group; 2021;12(1):1–6.

125. COVID-19 Case Investigation and Contact Tracing. Association of State and Territorial Health Officials. 2020 [cited 2021 Feb 4]; Available from: https://www.astho.org/ASTHOReports/COVID-19-Case-Investigation-and-Contact-Tracing-Considerations-for-Using-Digital-Technologies/07-16-20/

126. Loh P-S. Flipping the perspective in contact tracing. arXiv preprint arXiv:201003806. 2020;

127. NearForm; Solution for Covid-19 [Internet]. [cited 2020 Sep 30]. Available from: https://www.nearform.com/services/contact-tracing-app-development/

128. Troncoso et al. Decentralized Privacy-Preserving Proximity Tracing Version: 25 May 2020. [Internet]. [cited 2020 Sep 30]. Available from: https://github.com/DP-3T/documents/blob/master/DP3T%20White%20Paper.pdf

129. Pan-European Privacy-Preserving Proximity Tracing [Internet]. [cited 2020 Sep 30]. Available from: https://www.pepp-pt.org/

130. Centre for Disease Control Contact Tracing for COVID-19 [Internet]. [cited 2020 Nov 3]. Available from: https://www.cdc.gov/coronavirus/2019-ncov/php/contact-tracing/contact-tracing-plan/appendix.html#contact

131. Contact tracing: Public health management of persons, including healthcare workers, having had contact with COVID-19 cases in the European Union-second updateEuropean Centre for Disease Prevention and Control, 2020. ECDC Stockholm; 2020;

132. Qureshi Z, Jones N, Temple R, Larwood JP, Greenhalgh T, Bourouiba L. What is the evidence to support the 2-metre social distancing rule to reduce COVID-19 transmission. Accessed on: https://www cebm net/covid-19/what-is-the-evidenceto-support-the-2-metre-social-distancing-rule-to-reduce-covid-19-transmission. 2020;

133. Epidemiological Motivation of the Transmission Risk Level. Coronawarn Team, October, 2020 [Internet]. [cited 2021 Feb 4]. Available from: https://raw.githubusercontent.com/corona-warn-app/cwa-documentation/master/transmission_risk.pdf

134. Jehi L, Ji X, Milinovich A, Erzurum S, Rubin B, Gordon S, et al. Individualizing Risk Prediction for Positive Coronavirus Disease 2019 Testing. Chest S0012369220316548. 2020;

135. Munzert S, Selb P, Gohdes A, Stoetzer LF, Lowe W. Tracking and promoting the usage of a COVID-19 contact tracing app. Nature Human Behaviour. Nature Publishing Group; 2021;5(2):247–55.

136. Kretzschmar ME, Rozhnova G, Bootsma MC, van Boven M, van de Wijgert JH, Bonten MJ. Impact of delays on effectiveness of contact tracing strategies for COVID-19: a modelling study. The Lancet Public Health. Elsevier; 2020;5(8):e452–e459.

137. Yasaka TM, Lehrich BM, Sahyouni R. Peer-to-peer contact tracing: development of a privacy-preserving smartphone app. JMIR mHealth and uHealth. JMIR Publications Inc., Toronto, Canada; 2020;8(4):e18936.

138. Saw YE, Tan EY-Q, Liu JS, Liu JC. Predicting Public Uptake of Digital Contact Tracing During the COVID-19 Pandemic: Results From a Nationwide Survey in Singapore. Journal of medical Internet research. JMIR Publications Inc., Toronto, Canada; 2021;23(2):e24730.

139. How China Slowed Coronavirus: Lockdowns, Surveillance, Enforcers [Internet]. [cited 2020 Dec 3]. Available from: https://www.wsj.com/articles/how-china-slowed-coronavirus-lockdowns-surveillance-enforcers-11583868093

140. Lewis D. Contact-tracing apps help reduce COVID infections, data suggest. Nature. 2021;

141. Menges D, Aschmann HE, Moser A, Althaus CL, von Wyl V. The role of the SwissCovid digital contact tracing app during the pandemic response: results for the Canton of Zurich. medRxiv. Cold Spring Harbor Laboratory Press; 2021;

142. Ballouz T, Menges D, Aschmann HE, Domenghino A, Fehr JS, Puhan MA, et al. Digital proximity tracing app notifications lead to faster quarantine in non-household contacts: results from the Zurich SARS-CoV-2 Cohort Study. medRxiv. Cold Spring Harbor Laboratory Press; 2020;

143. Masel J, Shilen A, Helming B, Rutschman J, Windham G, Judd M, et al. Quantifying meaningful adoption of a SARS-CoV-2 exposure notification app on the campus of the University of Arizona. medRxiv. Cold Spring Harbor Laboratory Press; 2021;

144. The Epidemiological Impact of the NHS COVID-19 app [Internet]. [cited 2021 Mar 12]. Available from: https://github.com/BDI-pathogens/covid-19_instant_tracing/blob/master/Epidemiological_Impact_of_the_NHS_COVID_19_App_Public_Release_V1.pdf

145. Almagor J, Picascia S. Exploring the effectiveness of a COVID-19 contact tracing app using an agent-based model. Scientific reports. Nature Publishing Group; 2020;10(1):1–11.
