# Supplementary material for: Best Practice Guidance for Digital Contact Tracing Apps: A Cross-disciplinary Review of the Literature
Source: JMIR Mhealth Uhealth. 2021 Jun 7;9(6):e27753. doi: 10.2196/27753 (PMC8189288; doi:10.2196/27753)
Supplement: Multimedia Appendix 6 [file mhealth_v9i6e27753_app6.docx]

Appendix 6: Ethical Frameworks for Digital Contact Tracing Applications.

| Study | Framework components/considerations |
| --- | --- |
| Lo et al. 2020 [1] | Described a series of questions which should be answered yes to for a DCTA to be ethical:  1. Is the risk to public health serious?  2. Is the public health intervention effective for diminishing the public health risk?  3. Are the risk of the public health intervention acceptable? For exposure notification apps the following questions should be asked:  a) Is specific informed and voluntary consent required from the cellphone owner for the app to collect data and to notify potential contacts?  b) Are both the app user and potential contacts anonymous to each other?  c) Are the collected data the minimum needed to carry out an authorized public health purpose?  d) Is data use restricted to the public health purpose by designated public health officials? Is sharing data with other entities, such as law enforcement, immigration officials, the Department of Homeland Security, and commercial organizations expressly prohibited? Is combining the collected data with other data prohibited, so that individuals cannot be re-identified?  e) Are the data destroyed after a defined period, when they are no longer needed for the public health purpose?  f) Are strong security protections in place and tested?  g) Has the app been tested under field conditions?  4. Are the benefits and risks of the public health intervention equitably distributed?  5. Is the intervention the least restrictive alternative for achieving the public health goal? |
| Gasser et al. 2020 [2] | Described six ethical guiding principles:  1. Respect autonomy  2. Promote health beneficence  3. Promote justice  4. Prevent new infections and non-maleficence  5. Protect privacy  6. Support solidarity  Describes ethical and legal issues related to one or more of the six ethical principles:  1. Consent and voluntariness  2. Digital inequality  3. Expiration  4. Non-discrimination  5. Public benefit  6. Repurposing  7. Scientific validity  8. Systemic accountability  9. Transparency |
| World Health Organisation (2020) [3] | Described guiding principles:  1. Time limitation  2. Testing and evaluation  3. Proportionality  4. Data minimization  5. Use restriction  6. Voluntariness  7. Transparency and explainability  8. Privacy-preserving data storage  9. Security  10. Limited retention  11. Infection reporting  12. Notification  13. Tracking of COVID-19-positive cases  14. Accuracy  15. Accountability  16. Independent oversight  17. Civil society and public engagement |
| Morley et al. 2020 [4] | Described questions relating to principles and requirements:  **Principles: is this the right app to develop?**  1. Is it necessary?  2. Is it proportionate?  3. Is it sufficiently effective, timely, popular and accurate?  4. Is it temporary?  5. Is it proportionate?  **Requirements: is this app being developed in the right way?**  1. Is it voluntary?  2. Does it require consent?  3. Are the data kept private and users’ anonymity preserved?  4. Can users erase the data?  5. Is the purpose of data collection defined?  6. Is the purpose limited?  7. Is it used only for prevention?  8. Is it used for compliance?  9. Is it open-source?  10. Is it equally available?  11. Is it equally accessible?  12. Is there a decommissioning process? |
| Ranisch et al. 2020 [5] | Describes “substantive values”, “procedural values” and related guiding questions.  **Substantive values:**  1. Public health benefit  2. Harm minimisation  3. Privacy  4. Justice  5. Liberty/autonomy  6. Solidarity  7. Stewardship  **Procedural values:**  1. Transparency  2. Proportionality  3. General trustworthiness  4. Reasonableness  5. Accountability  6. Consistency  7. Engagement  8. Reflexivity |
| Ferretti et al. 2020 [6] | Described requirements for ethical digital contact tracing:  1. Oversight by an inclusive and transparent advisory board.  2. Agreement and publication of ethical principles by which the intervention will be guided.  3. Guarantees of equity of access and treatment.  4. Use of a transparent and auditable algorithm.  5. Integrating evaluation and research in the intervention to inform the effective management of future major outbreaks.  6. Careful oversight of and effective protections around the uses of data.  7. Sharing of knowledge with other countries, especially low- and middle-income countries.  8. Ensuring that the intervention involves the minimum imposition possible and that decisions in policy and practice are guided by three moral values: equal moral respect, fairness, and the importance of reducing suffering. |
| Parker et al. 2020 [7] | Discussed ethical considerations of DCTAs including:  1. Comparing benefits and harms of DCTAs versus harms of pandemic and a comparison with relative benefits and harms of other mooted options for non-pharmaceutical interventions to address harms of pandemic.  2. Can any infringement on privacy be justified in the context of the COVID-19 pandemic?  3. Balance between liberty and privacy with and without DCTAs.  4. Voluntariness  5. Data limitation  6. Equity, fairness, justice.  7. Consistency |

References

1. Lo B, Sim I. Ethical Framework for Assessing Manual and Digital Contact Tracing for COVID-19. Annals of Internal Medicine. American College of Physicians; 2020.

2. Gasser U, Ienca M, Scheibner J, Sleigh J, Vayena E. Digital tools against COVID-19: taxonomy, ethical challenges, and navigation aid. The Lancet Digital Health. Elsevier; 2020.

3. World Health Organization. Ethical considerations to guide the use of digital proximity tracking technologies for COVID-19 contact tracing: interim guidance, 28 May 2020. 2020.

4. Morley J, Cowls J, Taddeo M, Floridi L. Ethical guidelines for COVID-19 tracing apps. Nature Publishing Group; 2020.

5. Ranisch R, Nijsingh N, Ballantyne A, van Bergen A, Buyx A, Friedrich O, et al. Digital contact tracing and exposure notification: ethical guidance for trustworthy pandemic management. Ethics and information technology. Springer; 2020;1–10.

6. Ferretti L, Wymant C, Kendall M, Zhao L, Nurtay A, Abeler-Dörner L, Parker M, Bonsall D, Fraser C. Quantifying SARS-CoV-2 transmission suggests epidemic control with digital contact tracing. Science. 2020 May 8;368(6491):eabb6936. doi: 10.1126/science.abb6936. Epub 2020 Mar 31. PMID: 32234805; PMCID: PMC7164555.

7. Parker MJ, Fraser C, Abeler-Dӧrner L, Bonsall D. Ethics of instantaneous contact tracing using mobile phone apps in the control of the COVID-19 pandemic. Journal of Medical Ethics. Institute of Medical Ethics; 2020.
