# Supplementary material for: Best Practice Guidance for Digital Contact Tracing Apps: A Cross-disciplinary Review of the Literature
Source: JMIR Mhealth Uhealth. 2021 Jun 7;9(6):e27753. doi: 10.2196/27753 (PMC8189288; doi:10.2196/27753)
Supplement: Multimedia Appendix 7 [file mhealth_v9i6e27753_app7.docx]

Appendix 7: Evidence Supporting User Experience Considerations Recommendations.

Table 1: Evidence supporting universality recommendations.

| Universality aspects & Sources | Guidelines/Recommendations |
| --- | --- |
| **1 Accessibility**   - Academic literature [1][2][3][4][5][6] - Grey publications [10][11] - EU Regulations [12][13][30] - Guidelines [16][17][18] | App design accommodates the assistive technologies activated in the accessibility settings (e.g., screen readers) |
|  | App design accommodates a variety of input modalities, e.g., alternative options to interact with the app for individuals with without/or limited vision (e.g., external keyboard), with limited manipulation or strength (e.g., tangible devices), without/or limited hearing, without vocal capability |
|  | Provides text alternatives of videos, animations and audios, e.g., alternatives transcripts, captions and/or audio descriptions |
|  | Suggests a color-blind friendly design for users without perception of color:   - avoid poor color combinations: green-red, green-blue, green-brown, green-black, green-grey, blue-grey, light green-yellow, blue-purple - use of symbols to mark required form fields - underline the links - use of colors and symbols to convey error messages - use of patterns and textures to show the contrast in graphs and charts |
|  | Has simple features to make the technology easy to use from individuals with limited cognitive, language and learning abilities |
|  | Follows the accessibility guidelines for the design of the User Interface (UI) elements:   - navigational/interactive elements are easy to identify, they have clear, descriptive labels - input fields are easy to identify and fill in (e.g., voice control, QR code) - appropriate touch target size - appropriate contrast ratio for text and graphics - magnification up to 200% without loss of the UI characteristics |
|  | Accessible interactions   - provides onboarding pages for new users - supports portrait and landscape orientation without losing information - facilitates low physical effort when interacting with the UI (tasks without scrolling, interaction with one-hand use, the use of radio buttons and checkboxes) and gestures |
|  | Visibility   - shows when interactive elements are selected - provides feedback to the user input (the state of the system) - proposes feedback messages, error messages and alert notifications along with suggestions/guidelines to follow to correct the errors |
|  | Recoverability   - supports data entry error recovery - facilitates reversible actions |
| **2 Minors as Users**   - Academic literature [7][8] - Grey publications [10] - EU Regulations [14][15][30] - Guidelines [16] | Offers a notice for the suitability of the content |
|  | Supports parental control on the verification process (parental/legal guardian consent), contact tracing data collection and sharing with health authorities |
|  | Includes a simpler design for young users |
| **3 Cultural Universality**   - Academic literature [5] - Grey publications [10] - EU Regulations [12] - Guidelines [16] - DCTAs assessments [19] [20][21][22][23][24][25] | Supports many languages |
|  | Communicate/exchange encrypted Keys between various types of DCTAs (cooperation between different apps) |
| **4 Content**   - Academic literature [1][2] - Grey publications [10] - EU Regulations - Guidelines [16] | Avoid the use of jargon or acronyms related to clinical knowledge |
|  | Content retain the same after the adjustments are made to accommodate accessibility |
|  | Content accommodates the OS-level changes from the device settings (e.g., font, colour, contrast) |
| **5 Availability**   - Academic literature [9] - EU Regulations [30] - DCTAs assessments [19] [20][21][22][23][25] - Reviews available in Google/Apple stores [19] [26] | Internet access to download and set-up the application |
|  | Supports all platforms |
|  | Supports all types of mobile devices regardless of their screen sizes and resolutions |
|  | Works with foreign phone numbers (e.g., available to non-national accounts) |
| **6 Maintenance & Frequency of Upgrades**   - Grey publications [10] - Guidelines [16][30] - DCTAs assessments [19] [20][21][22][23][25] | App maintenance for effective functioning |
|  | Add new features |

Table 2: Evidence supporting user engagement recommendations.

| User engagement aspects & Sources | Guidelines/Recommendations |
| --- | --- |
| **1 Performance Feedback**   - Grey publications [10] - DCTAs assessments [19] [20][21] [22] [23][25] - Reviews available in Google/Apple stores [19] [26] | Updates on the daily reported cases (including new cases, deaths, hospital admissions) and their geographic areas (e.g., counties, towns) |
|  | High-risk areas (e.g., hot-spots) with a high concentration of confirmed COVID-19 cases (e.g., shops, parks, public transport) and safe travel zones (travelling information locally) |
|  | Number of active app users |
|  | Number of users reported positive COVID-19 diagnose |
|  | Intensive Care Unit (ICU): number of people that are currently in ICU, discharged from ICU and confirmed cases in ICU |
|  | Number of users reported positive COVID-19 diagnose and uploaded their digital contact tracing data |
|  | Number of people who were sent a close contact alert |
| **2 Helpfulness**   - EU Regulations [30] - Guidelines [16] - DCTAs assessments [19] [20][21][22][23][25] | Definitions, descriptions and examples |
|  | Help features, e.g., hotline, interactive assistance for technical support |
|  | Emergency call numbers for COVID-19 (e.g., buttons) to get urgent medical help from the emergency services |
| **3 Public Health Measures**   - DCTAs assessments [24] - Reviews available in Google/Apple stores [19] | Information on current government restrictions (e.g. national lockdown events) |
|  | Travel restrictions on crossing the border |
| **4 Educational Information**   - DCTAs assessments [19] [20][21][22][23][25] - Reviews available in Google/Apple stores [19] | Public health guidance, e.g., hand-hygiene instructions, mask-wearing instructions, distance, protection, meeting people |
|  | FAQ option |
|  | Monitoring:   - coronavirus testing - description of COVID-19 virus and its transmission - symptoms of COVID-19 - quarantine - recommendations for feeling unwell - vaccine (e.g., vaccination schedule, proof of vaccination, diary of daily symptoms tracking) |
| **5 Personal Information**   - Grey publications [10] - EU Regulations [29][30] - DCTAs assessments [19] [20][21] [22] [23][25] - Reviews available in Google/Apple stores [19] | Use forms of encryption for preserving personal data of the user, e.g., protect anonymity |
|  | Alert messages for informing user:   - the app is running in the back of the device - detected close contacts with other app users, e.g. less than 2m distance, prolonged more than 15 minutes - deactivated contact tracing function (e.g., turn on Bluetooth, GPS, microphone technologies to activate the function) - deleted collected contact tracing data (after 14 days) - daily check-in symptoms related to COVID-19 - washing hands |
|  | Visualizations:   - history of check-in symptoms (e.g., selected COVID-19 related symptom per day/week) - number of user’s close contacts with other app users per day(s)/week(s) (e.g., a counter on screen, bar charts) |
| **6 Personalisation & Control**   - Grey publications [10] - EU Regulations [16][29] [30] - DCTAs assessments [19] [20][21][22][23][25] - Reviews available in Google/Apple stores [19] | Tailor app aspects to the user preferences & Control over contact tracing function, e.g., feature to activate/deactivate at any time and environments |
|  | Tailor app aspects to the user preferences & Control over vaccination feature (e.g., activate/deactivate a digital vaccine passport) |
|  | Tailor app aspects to the user preferences & Control over alert messages and notifications, e.g., the app has built-in notification settings for quick access and activation/deactivation options |
|  | Control over the process of reporting positive covid-19 test |
|  | Control over generated anonymized contact tracing data:   - Easy access to the collected contact tracing data - Data manipulation, e.g. retrieve, access and delete - Upload and share the data with health authorities |
| **7 Time & Human Effort**   - Academic literature [4] - Guidelines [16][30] - DCTAs assessments [19] [20][21][22] [23][25] | Less time and human effort to complete the tasks |
| **8 Flexibility/Multimodality**   - Academic literature [3] - Guidelines [16][30] - DCTAs assessments [19] [20][21] [22][23][25] | Multiple modes for the execution of the tasks in various environments |
| **9 Multi-Tasking**   - Guidelines [16][30] - DCTAs assessments [19] [20][21] [22][23][25] | Supports simultaneous use with other devices/applications that also use Bluetooth (e.g. earphones, handsfree in cars), GPS, microphone |

References

1. M. Ballantyne, A. Jha, A. Jacobsen, J. Scott Hawker, and Y. N. El-Glaly, “Study of accessibility guidelines of mobile applications,” in *ACM International Conference Proceeding Series*, Nov. 2018, pp. 305–315, doi: 10.1145/3282894.3282921.

2. C. N. Harrington, L. Ruzic, and J. A. Sanford, “Universally Accessible mHealth Apps for Older Adults: Towards Increasing Adoption and Sustained Engagement,” in *International Conference on Universal Access in Human-Computer Interaction*, 2017, pp. 3–12, doi: 10.1007/978-3-319-58700-4_1.

3. L. R. Kascak, C. B. Rebola, and J. A. Sanford, “Integrating Universal Design (UD) principles and mobile design guidelines to improve design of mobile health applications for older adults,” in *Proceedings - 2014 IEEE International Conference on Healthcare Informatics, ICHI 2014*, Mar. 2014, pp. 343–348, doi: 10.1109/ICHI.2014.54.

4. F. A. Kasali, O. O. Taiwo, I. O. Akinyemi, O. B. Alaba, O. Awodele, and S. O. Kuyoro, “An Enhanced Usability Model for Mobile Health Application,” vol. 17, no. 2, pp. 20–29, 2019, [Online]. Available: https://sites.google.com/site/ijcsis/.

5. E. Kaur and P. D. Haghighi, “A context-aware usability model for mobile health applications,” in *ACM International Conference Proceeding Series*, Nov. 2016, pp. 181–189, doi: 10.1145/3007120.3007135.

6. D. Alonso-Ríos, A. Vázquez-García, E. Mosqueira-Rey, and V. Moret-Bonillo, “Usability: A critical analysis and a taxonomy,” *Int. J. Hum. Comput. Interact.*, vol. 26, no. 1, pp. 53–74, Jan. 2010, doi: 10.1080/10447310903025552.

7. N. Soni, A. Aloba, K. S. Morga, P. J. Wisniewski, and L. Anthony, “A framework of Touchscreen interaction design recommendations for children (TIDRC): Characterizing the gap between research evidence and design practice,” in *Proceedings of the 18th ACM International Conference on Interaction Design and Children, IDC 2019*, Jun. 2019, pp. 419–431, doi: 10.1145/3311927.3323149.

8. D. L. Gelman, *Design for Kids: Digital Products for Playing and Learning*. 2014.

9. S. Goel, R. Nagpal, and D. Mehrotra, “Mobile Applications Usability Parameters: Taking an Insight View,” in *Information and Communication Technology for Sustainable Development*, vol. 9, Mishra D., Nayak M., and Joshi A, Eds. Springer, Singapore, 2018, pp. 35–43.

10. P. Sanwikarja, “Contact tracing: How do you design an app millions of people will trust?,” *uxdesign.cc*, 2020. https://uxdesign.cc/how-do-you-design-an-app-millions-of-people-will-trust-8a63f5a5660a (accessed Jul. 02, 2020).

11. R. STAATS, “Designing UI with Color Blind Users in Mind - Secret Stache Media.” https://www.secretstache.com/blog/designing-for-color-blind-users/ (accessed Jul. 26, 2020).

12. ETSI, “ETSI EN 301 549 - V2.1.2 - Accessibility requirements for ICT products and services,” vol. 2, pp. 1–152, 2018, Accessed: Jul. 17, 2020. [Online]. Available: https://www.etsi.org/deliver/etsi_en/301500_301599/301549/02.01.02_60/en_301549v020102p.pdf.

13. EU, “DIRECTIVE (EU) 2016/2102 OF THE EUROPEAN PARLIAMENT AND OF THE COUNCIL of 26 October 2016 on the accessibility of the websites and mobile applications of public sector bodies,” *Off. J. Eur. Union*, vol. 327, pp. 1–15, 2016, [Online]. Available: https://eur-lex.europa.eu/eli/dir/2016/2102/oj.

14. General Data Protection Regulation (GDPR), “Art. 8 GDPR - Conditions applicable to child’s consent in relation to information society services,” *GDPR.eu*, 2018. https://gdpr-info.eu/art-8-gdpr/ (accessed Oct. 01, 2020).

15. G. Berman, K. Carter, M. García-Herranz, and V. Sekara, “Digital contact tracing and surveillance during COVID-19 General and Child-specific Ethical Issues,” 2020. Accessed: Jun. 16, 2020. [Online]. Available: www.ohchr.org/en/NewsEvents/Pages/DisplayNews.aspx?NewsID=25729&LangID=E.

16. Xcertia, “Xcertia mHealth App Guidelines,” *Xcertia*, 2019. https://www.himss.org/sites/hde/files/media/file/2020/04/17/xcertia-guidelines-2019-final.pdf.

17. “Mobile Accessibility: How WCAG 2.0 and Other W3C/WAI Guidelines Apply to Mobile.” https://www.w3.org/TR/mobile-accessibility-mapping/ (accessed Sep. 20, 2020).

18. w3cdevs, “Web Content Accessibility Guidelines (WCAG) Overview | Web Accessibility Initiative (WAI) | W3C,” *W3C.Org*. 2019, Accessed: Jan. 08, 2021. [Online]. Available: https://www.w3.org/WAI/standards-guidelines/wcag/.

19. HSE, “Covid Tracker App - HSE.ie,” *hse.ie*, 2020. https://www.hse.ie/eng/services/news/newsfeatures/covid19-updates/covid-tracker-app/covid-tracker-app.html (accessed Jun. 30, 2020).

20. Aman, “Aman,” 2020. https://amanapp.jo/en (accessed Jan. 07, 2021).

21. Open-Source Project Corona-Warn-App, “Corona-Warn-App,” 2020. https://www.coronawarn.app/en/ (accessed Jun. 29, 2020).

22. novid.org, “NOVID,” 2020. https://www.novid.org/#howitworks (accessed Jun. 16, 2020).

23. Path Check Foundation, “Home - COVID Safe Paths | COVID Safe Paths,” *covidsafepaths.org*, 2020. https://www.pathcheck.org/ (accessed Jun. 27, 2020).

24. Gov.pl, “STOP COVID - STOP COVID,” 2020. https://www.gov.pl/web/protegosafe (accessed Jan. 12, 2021).

25. C. Storni *et al.*, “Toward a Compare and Contrast Framework for COVID-19 Contact Tracing Mobile Applications : a Look at Usability,” 2021, accepted for the HEALTHINF2021 conferennce

26. K. Rekanar *et al.*, “Sentiment Analysis of User Feedback on the HSE Contact Tracing App,” 2020, doi: 10.21203/rs.3.rs-96174/v1.

27. J. Buckley *et al.*, “A Proposal for the SFI Covid-19 Rapid Response Funding Call,” 2020. Accessed: Sep. 15, 2020. [Online]. Available: https://tinyurl.com/y4qbvk8c.

28. T. Welsh *et al.*, “Towards a Taxonomy for Evaluating Societal Concerns of Contact Tracing Apps,” in *In 2020 7th International Conference on Behavioral, Economic, and Socio-Cultural Computing (BESC)*, 2020.

29. ICO, “COVID-19 Contact tracing: data protection expectations on app development,” 2020. Accessed: Jun. 15, 2020. [Online]. Available: https://ico.org.uk/media/for-organisations/documents/2617676/ico-contact-tracing-recommendations.pdf.

30. eHealth Network E. Mobile Applications to support contact tracing in the EU’s fight against COVID-19. Common EU Toolbox for Member States. 2020;1–56.
